# Novel Peptidomic Approach for Identification of Low and High Molecular Weight Tauopathy Peptides Following Calpain Digestion, and Primary Culture Neurotoxic Challenges

**DOI:** 10.3390/ijms20205213

**Published:** 2019-10-21

**Authors:** Hamad Yadikar, Connor Johnson, Niko Pafundi, Edwin Mouhawasse, Lynn Nguyen, Isabel Torres, Milin Kurup, Zhihui Yang, Firas Kobeissy, Richard Yost, Kevin K. Wang

**Affiliations:** 1 Program for Neurotrauma, Neuroproteomics & Biomarkers Research, Departments of Emergency Medicine, Psychiatry, Neuroscience and Chemistry, University of Florida, Gainesville, FL 32611, USA; connorj96@ufl.edu (C.J.); nikop1997@ufl.edu (N.P.); emouhawasse@ufl.edu (E.M.); lynnng@ufl.edu (L.N.); torresi@ufl.edu (I.T.); milinkurup@ufl.edu (M.K.); zhihuiyang@ufl.edu (Z.Y.); firasko@gmail.com (F.K.); 2 Department of Biological Sciences, Faculty of Science, Kuwait University, P.O. Box 5969, Safat 13060, Kuwait; 3 Department of Chemistry, Chemistry Laboratory Building, University of Florida, Gainesville, FL 32611, USA; ryost@ufl.edu; 4 Faculty of Medicine, American University of Beirut Medical Center, Beirut F32611, Lebanon; 5 Brain Rehabilitation Research Center, Malcom Randall VA Medical Center, 1601 SW Archer Rd. Gainesville, FL 32608, USA

**Keywords:** tau proteolysis, tauopathy, calpain, peptidomics, neurodegeneration

## Abstract

Tauopathy is a class of a neurodegenerative disorder linked with tau hyperphosphorylation, proteolysis, and aggregation. Tau can be subjected to proteolysis upon calpain activation in Alzheimer disease (AD), and traumatic brain injury (TBI). We and others have extensively researched calpain-mediated tau breakdown products (Tau-BDP; 45K, 35K, and 17K). Tau proteolysis might also generate low molecular weight (LMW ≤10K) proteolytic peptides after neurodegenerative damage. In this study, we have subjected purified tau protein (phospho and non-phospho) and mouse brain lysate to calpain-1 digestion to characterize the LMW generated by nano-liquid chromatography coupled to electrospray ionization to tandem mass spectrometry (nano-LC-ESI-MS/MS). We have also challenged differentiated primary cerebrocortical neuronal cultures (CTX) with neurotoxic agents (calcium ionophore calcimycin (A23187), staurosporine (STS), N-methyl-D-aspartate (NMDA), and Maitotoxin (MTX)) that mimic neurodegeneration to investigate the peptidome released into the conditioned cell media. We used a simple workflow in which we fractionate LMW calpain-mediated tau peptides by ultrafiltration (molecular weight cut-off value (MWCO) of 10K) and subject filtrate fractions to nano-LC-MS/MS analysis. The high molecular weight (HMW) peptides and intact proteins retained on the filter were analyzed separately by western blotting using total and phospho-specific tau antibodies. We have identified several novel proteolytic tau peptides (phosphorylated and non-phosphorylated) that are only present in samples treated with calpain or cell-based calpain activation model (particularly N- and C-terminal peptides). Our findings can help in developing future research strategies emphasizing on the suppression of tau proteolysis as a target.

## 1. Introduction

Microtubule-associated protein tau (MAPT) is a soluble protein whose function is to control the stability of the axonal microtubules [1]. Tau is expressed in the central nervous system (CNS) and mainly present in neurons and at lower levels in astrocytes and oligodendrocytes [1]. Tauopathies consist of abnormal post-translational modifications of tau that leads to the formation of tau oligomers, pair helical filaments, and neurofibrillary tangles. Tauopathies of the CNS, including Alzheimer Disease (AD), chronic traumatic encephalopathy (CTE), and Parkinson’s disease, are associated with defective tau protein that can no longer stabilize the microtubule [2]. Processes occurring in tauopathies disrupt tau physiological function of microtubule stabilization and formation [3,4,5]. Examples of post-translational modifications (PTMs) that occur in tauopathies include hyperphosphorylation, acetylation, deamination, glycation, glycosylation, isomerization, methylation, nitration sumoylation, ubiquitination, and proteolysis [6]. Phosphorylation is one of the most well-characterized and researched PTM of tau, for its direct ability to disrupt binding to microtubules and contribution to aggregation, as well as for neurofibrillary tangles (NFT) formation [7]. 

Tau proteolysis has generated a considerable amount of research interest because of its involvement with tauopathies, mainly through calpain activation [8]. Calpain family consists of 14 different-cysteine-dependent proteases that are regulated by calcium binding to a specific conserved site in all members of the family [9]. Tau proteolysis by calpain-1 and 2 contribute to opposing functions of regulating synaptic plasticity and neurodegeneration [9,10,11,12]. Calcium-mediated proteolytic cleavage of tau by calpain can lead to the production of truncated tau break-down products (tau-BDP) which are susceptible to hyperphosphorylation and aggregation processes. Calpain can degrade a wide variety of cytoskeletal proteins, such as microtubule-associated protein 2 (MAP2) and αII-spectrin [9,11,13].

Calpain-mediated αII-spectrin cleavage products are used to detect post-TBI axonal injury and neurodegeneration following TBI [14]. Calpain degradation of tau produces a 35K tau fragment and a 17K tau fragment that can contribute to neurotoxic events within the brain cells [15]. In AD tauopathy, increased calpain activity and decreased levels of endogenous calpain inhibitor, calpastatin, have been reported [16]. A study of 17K tau-BDP have shown the decreased microtubule-binding ability of tau and increased susceptibility to accumulation in the perikarya [15]. Another study showed that amyloid-β (Aβ) could activate calpain in cultured hippocampal neurons, producing the 17K tau-BDP and cause neurodegeneration properties, which are delayed by inhibition of calpain activation [17]. Calpain is also an upstream activator of the extracellular-regulated kinase (ERK), which can phosphorylate tau protein [18]. Increase in ERK expression leads to early accumulation of tau in neurons and glia in various tauopathies [19]. 

Most proteomics studies nowadays follow the bottom-up approach, where protein samples are digested with trypsin, followed by different fractionation techniques for peptide identification. This approach results in the generation of tryptic peptides and leads to fragmentation of native endogenous peptides present in the samples; thus, making their identification a challenge. Although structural and functional studies of tau protein have been researched extensively in the past, studies characterizing the endogenous low molecular weight (LMW, ≤10K) tau peptides are of interest due to their association with a disease state. These natural peptides could reflect several active processes occurring in vivo in the human brain, such as enzymatic/proteolytic activity, exocytosis, and tau aggregation [6,20]. The standard analytical proteomics platforms use trypsin digestion and produce tryptic peptides that can be used for quantification using mass spectrometric analysis. One of the significant disadvantages of proteomic studies is the loss of valuable natural peptide information present in the biological samples leading to the generation of artificial tryptic peptides ending with Lysine and Arginine residues. This might be useful for increasing the identification and proteome coverage given the abundance of Lysine and Arginine residues in a particular protein. However, the naturally released peptides following TBI and other neurodegenerative diseases provide valuable use for biomarker studies that can be applied in diagnosis and therapy “theranostic”. This is defined as peptidomics, and any peptidomic approach involves studying the entire set of naturally occurring endogenous peptides in biological samples. To our knowledge, there are no peptidomic studies performed systematically to identify low molecular weight tau peptides as biomarker candidates for neurodegenerative diseases. 

Thus, this study aimed to characterize tau peptides generated from calpain or under different cell-based neurotoxic conditions. To achieve this goal, we performed an in vitro calpain-1 digestion of purified human tau-441 (non-phosphorylated and phosphorylated) and naïve transgenic human tau (htau) mice to study intact or high molecular weight (HMW) and LMW tau products by western blotting and nano-liquid chromatography coupled to electrospray ionization and tandem mass spectrometry (nLC-ESI-MS/MS), respectively. We have also used non-phosphorylated and phosphorylated tau to survey for unique LMW peptidome generated by calpain-1. To take this a step further, we have used rat primary cerebrocortical neuronal culture (CTX) to study HMW and LMW tau fragments generated when challenged with neurotoxic conditions that trigger necrosis (MTX), apoptosis (STS), and calcium influx (A23187). Moreover, CTX culture was treated with okadaic acid (OA) to induce tau hyperphosphorylation, test the differential susceptibility of tau to proteolysis, and analyze the release of LMW proteolytic fragments in conditioned cell media. 

OA is well known to inhibit protein phosphatases 1/2A, which results in tau hyperphosphorylation within the afflicted cells [21]. Therefore, OA was used to serve as a tauopathy-relevant neuronal-based model. The processed biological samples were subjected to ultrafiltration devices with a molecular weight cut-off (MWCO) value of 10K. This method was used to enrich LMW fractions that were analyzed by nano-LC- tandem mass spectrometry. We detected novel proteolytic tau peptides that might be linked to neurodegeneration, as they were exclusively present in treatments but not in controls. We were also able to derive calpain-1 cleavage sites using our MS data. We found phosphorylated sites that that could be critical to the pattern of calpain-1 proteolysis of tau protein. Our findings might aid in developing promising future neurotherapeutic strategies with an emphasis on the suppression of tau proteolysis and biomarker candidates for neurodegenerative diseases.

## 2. Results and Discussions

**Figure 1:**
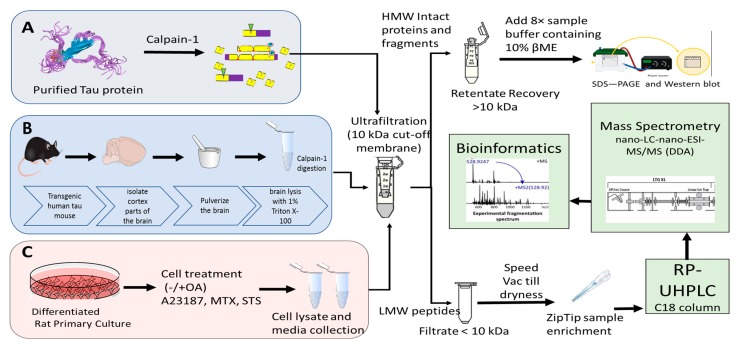
Peptidomic workflow for nano-liquid chromatography coupled to electrospray ionization and tandem mass spectrometry (nano-LC-ESI-MS/MS) for the analysis of purified Tau protein, rat differentiated primary culture and mouse brain lysate. Subjecting the proteins to an in vitro calpain-1 digestion results in both high molecular weight (HMW) protein fragments and low molecular weight (LMW) proteolytic peptides production. HMW fragments were reduced with 10% β-mercaptoethanol (βME) and 8× sample buffer. The LMW fragments were subjected to C_18_ zip-tip and analyzed using reverse-phase- ultra-high-pressure chromatography (RP-UHPLC) followed by nano-LC-ESI-MS/MS with a data-dependent acquisition (DDA). The HMW fragments were validated by western blotting. Panel (**A**) shows the workflow followed for proteolysis of recombinant purified tau-441 (non-phosphorylated and phosphorylated). Panel (**B**) shows the isolation of cortex fraction of transgenic human tau (htau) mouse brain. Panel (**C**) shows the protocol followed for the treatment of rat cerebrocortical primary neuronal culture with neurotoxic agents. The neurotoxic agents were used includes okadaic acid (OA), staurosporine (STS), A23187, and maitotoxin (MTX).

Tau proteolytic peptides are promising biomarkers for Alzheimer disease (AD), traumatic brain injury (TBI), and chronic traumatic encephalopathy (CTE). In this study, we designed a simple protocol to collect and enrich the high and low molecular weight (HMW and LMW) tau fragments that were generated in vitro and in vivo using a proteolytic enzyme or neurotoxic agents that mimic the conditions of neurodegenerative diseases. In the first step of this workflow, calpain-1 was added to purified tau-441 (non-phosphorylated and phosphorylated) protein and transgenic mouse brain lysate (Figure 1A,B). We have included the non-phosphorylated (tau) and phosphorylated purified tau-441 (p-tau) protein in our study to account for the differential proteolytic peptides that can be generated by calpain digestion.

The neurotoxic agents were used (staurosporine (STS), calcium ionophore (A23187), and maitotoxin (MTX)) on a differentiated rat primary cerebrocortical neuronal culture in the presence and absence of okadaic acid (OA) (Figure 1C) to characterize the proteolytic peptides generated under tauopathy neurodegenerative conditions [22,23,24]. All samples were subjected to ultrafiltration device with a molecular weight cut-off value of 10K. The retentate fractions (≥10K) were analyzed by sodium dodecyl sulfate-polyacrylamide gel electrophoresis (SDS-PAGE)/Western blotting, and the filtrate fractions were subjected to nano-LC-MS/MS analysis (Figure 1C). 

### 2.1. Immunoblot Validation of Human Calpain-Mediated Tau In Vitro Digestion

**Figure 2:**
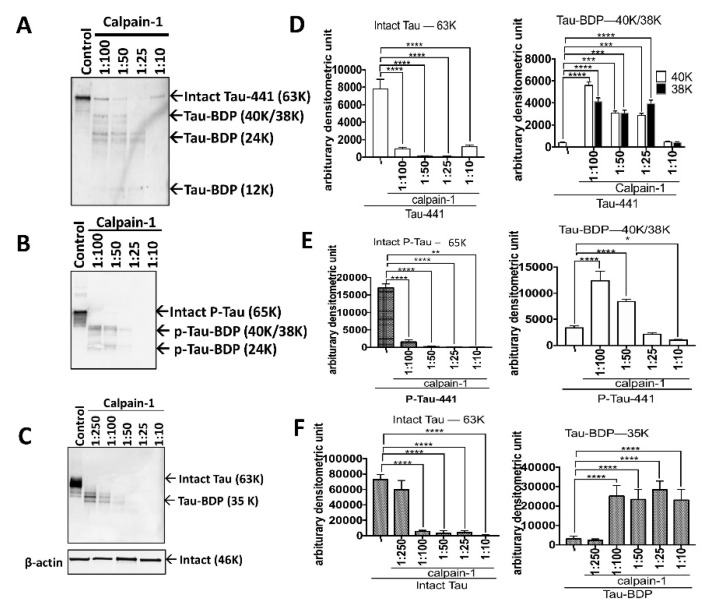
Identification of high molecular weight calpain-mediated tau proteolytic peptides by immunoblotting. (**A**) (recombinant tau), (**B**) (recombinant phospho-tau), and (**C**) (mouse brain lysate) are Western blots displaying intact tau and high-molecular-weight tau breakdown products (HMW-tau-BDPs) treated with different calpain-1 concentrations (1:100, 1:50, 1:25, and 1:10). (**D**–**F**) are quantification graphs of the intact tau (63–65K) and tau-BDP (40K/38K) from tau, p-tau, and mouse brain lysate, respectively. Total tau antibody (DA9, amino acids (a.a.): 102–145) was used. Densitometric quantification of the intact and tau-BDP was performed using image-J. Data are presented as ± standard error of the mean (SEM) for *n* = 3. Statistical analysis was performed with one-way analysis of variance (ANOVA). For multiple comparisons, one-way ANOVA followed by the Bonferroni’s post-hoc test was performed. * *p* < 0.05, ** *p* < 0.01, *** *p* < 0.001, **** *p* < 0.0001, and ns: non-significant.

Recombinant human tau and p-tau (441-residue; 4R isoform) were digested with calpain-1 in the ratios of 1:100, 1:50, 1:25, and 1:10 (Figure 2A,B,D,E). A few proteolytic fragments of tau and p-tau were observed with Coomassie Brilliant Blue staining (Figure S1). Following the ultrafiltration of samples, the retentate fractions (>10K) were subjected to SDS-PAGE followed by Western Blotting. Control samples showed intact tau at 63K (Figure 2A) and intact p-tau at 65K (Figure 2B) using total tau DA9 antibody (a.a. 102–145). Addition of varying concentration of calpain-1 caused a significant reduction (*p* < 0.0001) of tau-63K and p-tau-65K (Figure 2A,B,D,E). Moreover, tau break-down products (Tau-BDP) 40K, 38 K, and 24K elevated significantly (*p* < 0.0001) with increasing concentration of calpain-1 (Figure 2A,D). In a previous study, p-tauBDP-24K have been detected with an antibody that binds the N-terminal NH_2_-PTREPKKVAVV suggesting a cleavage site between Gly157/Ala158 of the full-length tau [25]. The tau-BDP-40K/38K and 24K bands were non-detectable with calpain 1:10 ratio, suggesting their vulnerability to proteolysis (Figure 2A).

To validate the fidelity of tau proteolysis in a more complex biological system, we further performed calpain-1 digestion in a mouse transgenic human tau (htau) cortex brain lysate (5 μg) and analyzed the samples by SDS-PAGE followed by western blotting (Figure 2C,F). Western blot analysis of retentate fractions showed that total intact tau (63K; DA9) was highly vulnerable to calpain-1 from brain lysate source. We observed a cluster of immunoreactive bands (tau-BDP-35K) when samples were probed with total tau DA9, DAKO (Agilent, Santa Clara, CA, USA) (amino acids: 243–441) and phospho-specific tau antibody RZ3 (pT231) (Figure 2C; Figure S1A,B). β-actin a loading control showed equal amounts of protein. Moreover, with DAKO antibody, we were able to observe a 12K tau-BDP that diminished gradually with increasing concentration of calpain in brain lysate samples (Figure S1A). These tau-BDPs have been reported in an earlier study [25]. We did not observe the tau-BDP-15K with DA9, RZ3, and CP13 (Figure 2C; Figure S1B,C). With this knowledge, we confirmed that tau from various sources is cleaved and fragments of multiple sizes are generated. The differences in the molecular weight breakdown products detected using immunoblotting between tau, p-tau, and brain lysate, suggest that there might be a differential fragmentation pattern of tau induced by calpain-1. Since the ratio of tau:calpain is a crucial factor that determines the protease activity, we experimented with equal protein content (10 μg per sample) and ran the samples in one blot to compare the amount of tau and proteolytic fragments generated (Figure S2).

### 2.2. nLC-ESI-MS/MS Analysis of Purified Tau and Mouse Brain Lysate

The tau, p-tau, and mouse brain lysate ultrafiltrate fractions were subjected to nano-LC-MS/MS analysis. We have used multiple treatments of calpain-1 (1:250, 1:100, 1:50, 1:25, and 1:10 enzyme to protein dilutions) to create a library of the different fragments that could potentially be generated from tau mimicking neurodegenerative environments. Identification of non-specific proteolytic peptides by sequence database is more challenging than the identification of peptides using a specific protease (e.g., trypsin). This is due to unpredictable enzyme cleavage specificity, cleavage by more than one protease in the biological mixture, and increased length of peptide sequences, thereby increasing the search time by a factor of 100–1000. To increase the confidence of our matches, we performed the database searches using false discovery rate (FDR) of 1%, searched against a decoy database, used minimally five peptide sequence matches (PSMs) based on MS/MS, and an XCorr value of 2. We identified peptides relating to the protein turnover. All peptides, which are found in control, were excluded from our bioinformatics analysis.

Moreover, since we are only interested in the pathogenesis of tau protein in this study, our database search included the different six isoforms of tau protein (based on Unitprot, Human Proteome Org and Human Protein Atlas organ distribution data sets). We also were able to identify exclusive tau peptide fragments that were only present in either tau, p-tau, or brain lysate, but not in all fractions (Table 1, Table 2, Table 3 and Table 4). 

**Figure 3:**
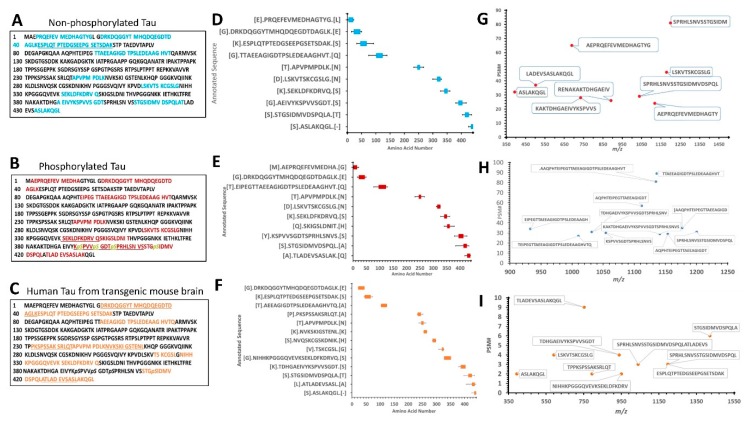
Characterization of low molecular weight calpain-mediated tau proteolytic peptides by mass spectrometry. (**A**–**C**) panels show tau (P10636-8) sequence with colored residues representing the peptides identified from recombinant tau, recombinant p-tau, and mouse brain lysate tau, respectively. The underlined residues represent the overlapping sequence between two proteolytic peptides. Amino acid residues in black color represent an unidentified sequence. (**D**–**F**) are schematic representations of tau, p-tau, and mouse brain htau peptides generated from calpain-1 digestion, respectively. The core peptides are shown in a colored rectangular box, while the variability of the different lengths of peptides is represented as error bars. These error bars were calculated from all biological replicates peptides that were high in confidence, XCorr value of 3, and minimum PSM value of 20 from Sequest HT search engine of proteome discoverer 2.2. Peptides found in control were omitted. Brackets on y-axis between letters represent adjacent amino acids that are not part of the original sequence but might be necessary for calpain cleavage. (**G**–**I**) are top-scored peptides based on peptide sequence matches (PSMs) from tau, p-tau, and mouse brain lysate samples, respectively. The y-axis represents the count number of each peptide, and x-axis represents mass over charge ratio (*m/z*) for non-phosphorylated-tau-441. For each selected peptide, at least 20 PSM and an XCorr value of 3 was used from proteome discoverer 2.2 software to present high confidence identifications.

Figure 3A–C show the amino acid sequence of tau-441 isoform with the identified proteolytic peptides from recombinant tau, p-tau, and mouse brain highlighted, respectively. The identification of peptides from recombinant tau and p-tau had very similar sequences, while peptides from mouse brain differed noticeably. The p-tau samples had phosphorylated residues (highlighted in yellow) that were not present in either unphosphorylated tau and mouse brain htau (Figure 3B). Figure 3D–F show a schematic representation of the top 10 peptide sequence matches (PSM) with core peptide and the varying lengths represented as error bars, observed in tau, p-tau, and mouse brain lysate samples. The N-terminal region for all three samples showed resemblance in the pattern of fragmentation with non-phosphorylated tau showing slightly more vulnerability to proteolysis, suggesting the effect of phosphorylated residues to calpain digestion. It has been reported by Johnson et al. group that in CSF samples included in their study contained fragmented species which predominantly consisted of the N-terminus tau using epitope-specific antibodies [6]. 

For brain Lysate filtrate fractions, we have identified two clusters of N-terminal, the middle region, and C-terminal domain peptides with a fragmentation pattern that is again closely similar to the recombinant purified tau-441 calpain-mediated proteolysis pattern (Table 3; Figure 3C,F,I). The PSM around the region a.a.:413–441 of brain lysate considerably overlapped with the purified tau protein coverage (Figure 3D–F), suggesting that calpain-1 digested the cellular htau similar to purified htau protein. However, the mid-region (a.a. 225–300) of brain lysate showed a different fragmentation pattern of tau (Figure 3F). Figure 3G–I summarize the top 10 highest peptide sequence matches (PSM) scores of calpain-mediated tau proteolytic peptides from recombinant tau and brain lysate source. The PSM number represent the times the Mass spectrometric instrument detected the fragments based on the data-dependent acquisition. The purified recombinant tau samples had more peptides counts (80–100 peptides) compared to the mouse brain sample (10 peptides) (Figure 3G–I). A possible explanation is that calpain can proteolyze other proteins as a substrate in a brain sample decreasing its potency to tau compared to the samples that have tau only. 

**Figure 4:**
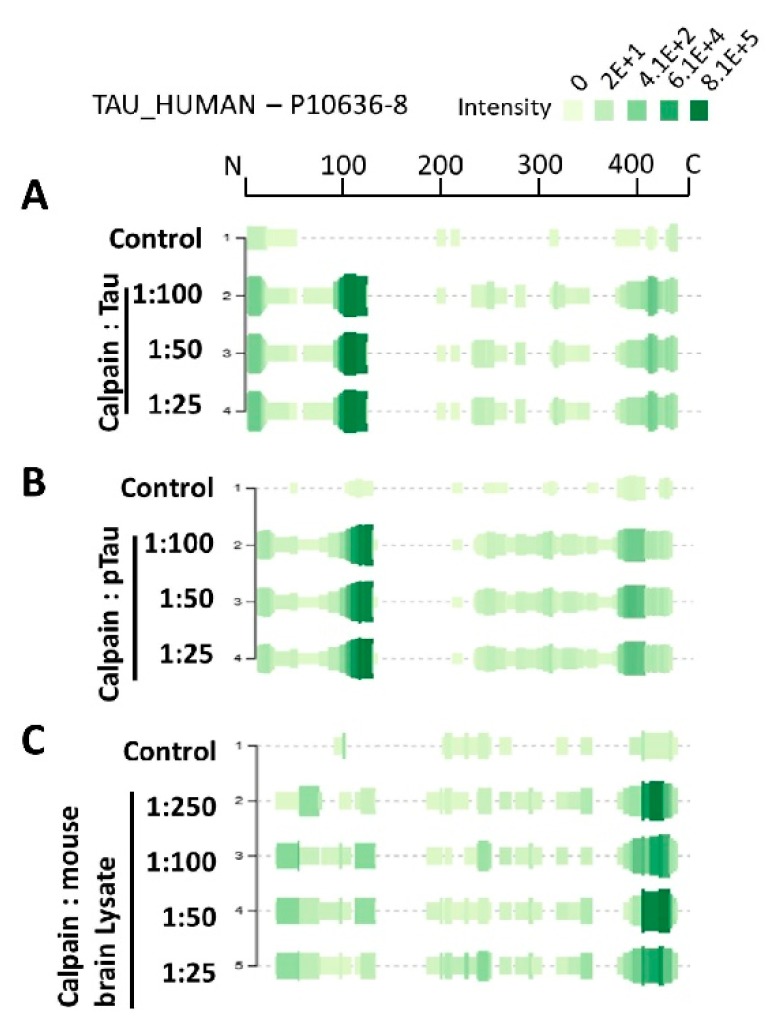
Tau proteolytic peptides heatmap profile from samples digested by calpain. (**A**–**C**) shows mass spectrometric intensities peptide profile of calpain-mediated tau, p-tau, and mouse brain, respectively (accession#: P10636-8). At each amino acid residue along the protein, the height of the green bars is proportional to the count of peptides overlapping this position. The intensity of the color (green) is proportional to the sum of the peptide intensities overlapping this position. The left side of the plots shows the dilution of calpain used for each condition.

A well-known approach for monitoring peptide abundance for peptidomics studies is implementing the use of peak intensity. Peak intensity involves the calculation of the ion current of the peptide as it elutes from LC/MS usually integrating signal over the entire elution period, measuring the peak value. Figure 4A–C demonstrate the peptidome profile of purified human tau, p-tau, and mouse brain lysate digested by calpain-1, respectively, based on their mass spectrometric intensity. At each amino acid residue along the protein, the height of the green bars is proportional to the PSM count of peptides overlapping this position. The intensity of the color (green) is proportional to the sum of the peptide intensities overlapping this position. The proteolytic peptide profile of tau and p-tau shows a similar distribution of count and intensity (around a.a. 100–130). Interestingly, the brain tau peptides showed the highest peak intensity around the C-terminal region. Such differences might be due to tau tertiary/quaternary structure complexity causing different patterns in calpain digestion. 

**Figure 5:**
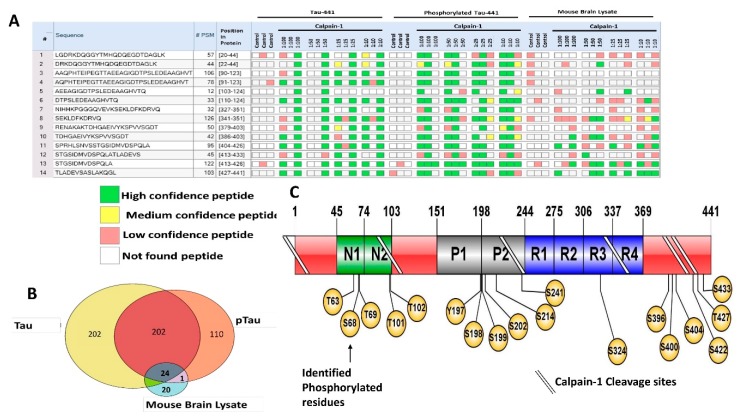
Composite summary of calpain-mediated tau proteolytic peptides and phosphorylation sites. (**A**) A spreadsheet showing calpain-mediated proteolytic peptides identified from Tau, p-tau, and mouse brain lysate ultrafiltrate samples. This table listing shows the confidence of each peptide in each fraction. Not found peptide is represented as white. Green represents high confidence peptides. Yellow represents medium confidence peptides. Red represents low confidence peptides. Each box represents a biological replicate, and each experiment was done at least *n* = 3. (**B**) Venn diagram showing the total number of peptides released in each of the biological replicates from the different samples. (**C**) A schematic representation showing the domains of tau protein and displaying the location of phosphorylation identified by nano-LC/MS/MS and predicted calpain cleavage sites derived from in this study. All tables and diagrams created using the proteome discoverer 2.2.

Of particular interest, as biomarkers are the C-terminal domain tau peptides that were found in all samples such as SPRHLSNVSSTGSIDMVDSPQLA (a.a. 404–426), STGSIDMVDSPQLA (a.a. 413–426), and TLADEVSASLAKQGL (a.a. 427–441) (Figure 5A). From our bioinformatics analysis (Sequest HT), we identified a total of 404 peptides from tau, 312 from p-tau and 45 peptides from brain lysate. Twenty-four peptides were in common between the three types of samples (*n* = 3) (Figure 5B). Around 110 peptides were found only in p-tau samples, 202 peptides in tau samples, and 20 peptides from brain lysate. As for the phosphorylation sites identified with mass spectrometry, we were able to identify new sites on the p-tau that are not found in the tau, particularly close to the C-terminal domain including Ser 400, Thr 403, Ser 412, and Tyr 394 (Table 3; Figure 5C). These novel phosphorylation sites might cause differential calpain cleavage of tau producing unique peptides that are exclusively found in phosphorylated tau fractions. The unique phosphorylation sites might cause calpain to process tau differently generating the various LMW peptides.

**Figure 6:**
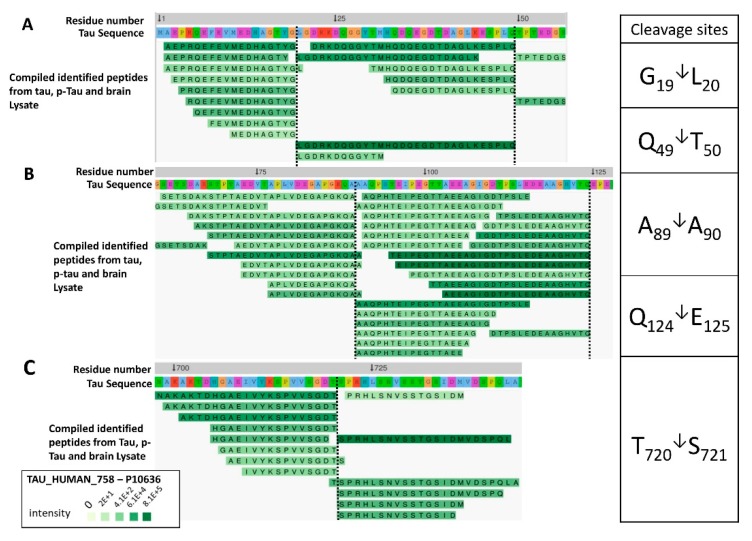
View of a customized Peptigram peptide alignment map compiled from tau, p-tau, and mouse brain lysate. The peptides were aligned to fit the longest human tau precursor protein (accession #: P10636; 758 a.a.). The representative peptides aligned were obtained by filtering and compiling peptides identified from tau, p-tau, and brain lysate digested by calpain-1. The black horizontal dotted lines display the predicted calpain cleavage sites which are summarized on the table to the right of the figure. The alignments represented are retrieved by ProViz and were further modified [26]. Peptides from the input data are highlighted in green color. The intensity of each peak detected by mass spectrometry is represented by a green color gradient, the darker the green color the higher the intensity. (**A**) This alignment is generated from tau-758 starting from the N-terminal domain: a.a. 1–57 (**B**) covers amino acid 60–128 and (**C**) cover amino acid 698–744.

Figure 6 shows a Peptigram of longest tau isoform (P10636; a.a. 758) identified peptides alignment map generated from ProViz web-based visualization tool [26]. The map alignment was created using a compiled excel file that contains the highest-ranked calpain-mediated peptides (total of 50 peptides) from tau, p-tau, and brain lysate to determine the potential cleavage sites of calpain. A cluster of varying lengths of the peptides AEPRQEFEVMEDHAGTYG (a.a. 2–19) and LGDRKDQGGYTMHQDQEGDTDAGLK (a.a. 20–44) has been identified with high confidence, suggesting a cleavage site between G19/L20, spanning the N-terminal region of tau (Figure 6A). The tau fragments 26–44, 26–44, 1–44 and 45–441 have been reported as calpain-1-mediated fragments causing N-methyl-D-aspartate receptor (NMDAR)-mediated cell death in cerebellar granule cells [6]. Moreover, a cluster of varying lengths of the peptides SETSDAKSTPTAEDVTAPLVDEGAPGKQA (a.a. 61–89) and AAQPHTEIPEGTTAEEAGIGDTPSLE (a.a. 90–115) has been found with high confidence, suggesting a cleavage site between A89/A90 (Figure 6B). The peptides spanning the C-terminal domain RENAKAKTDHGAEIVYKSPVVSGDT (a.a. 696–720) and SPRHLSNVSSTGSIDMVDSPQLATLADEVS (a.a. 721–750). Predominant fragments detected by our nano-LC-MS/MS analysis are the C-terminal peptides SPRHLSNVSSTGSIDMVDSPQLATLADEVS (a.a 404–433) with *m/z* 1038.75 and TLADEVSASLAKQGL (a.a. 427–441) suggesting a cleavage site T720/S721 (Figure 6C).

### 2.3. Analysis of Tau Fragments in Differentiated Rat Primary Cerebrocortical Neurons Subjected to Neurotoxic Conditions

**Figure 7:**
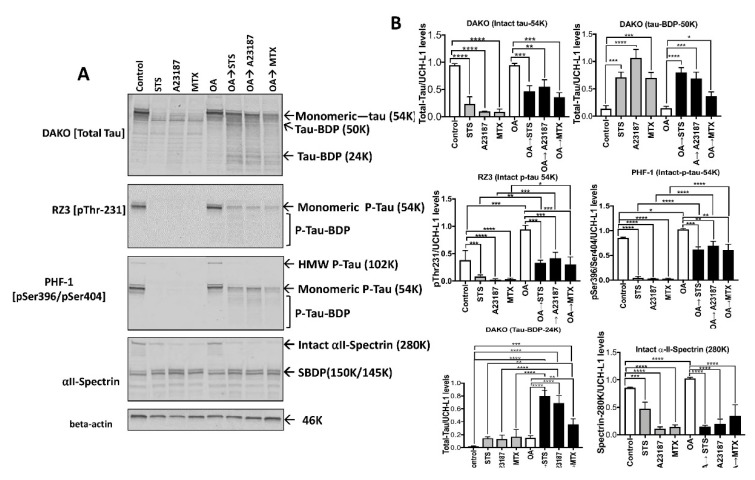
Basal and okadaic acid (OA)-induced tau proteolysis following neurotoxin challenges in differentiated rat primary cerebrocortical neurons. Basal and OA-induced tau proteolysis following neurotoxin challenges in rat cerebrocortical neurons. (**A**) Rat cerebrocortical cultures (CTX) were either untreated (control) or pre-treated with a phosphatase inhibitor (OA, 100 nM) for 16 h. For tau proteolysis analysis, cultures were treated with neurotoxin agents including STS (0.5μ M), A23187 (20 μM) and MTX (10 nM). After the treatment, cell lysates were harvested for protein and immunoblotting analysis with total tau monoclonal antibody (DAKO; top panel), phospho-tau antibodies (RZ3 and PHF-1), and αII-spectrin monoclonal antibody. The beta-actin antibody was used as a loading control. Results are representative of three independent experiments. (**B**) Show densitometric quantifications from the immunoblots. Data are presented as ± SEM for *n* = 3. Statistical analysis was performed with one-way ANOVA. For multiple comparisons, one-way ANOVA followed by the Bonferroni’s post-hoc test was performed. * *p* < 0.05, ** *p* < 0.01, *** *p* < 0.001, **** *p* < 0.0001, and ns: non-significant.

To test tau vulnerability to calpain or caspase in a system that represents the sophisticated structure of the CNS, we used neurotoxic agents on the differentiated rat primary cerebrocortical culture (CTX) (Figure 7A,B). We added pro-necrotic calcium ionophore A23187, pro-apoptotic staurosporine (STS), calcium ionophore maitotoxin (MTX) for 16h, with or without okadaic acid (OA; 6h) in CTX culture to mimic intracellular fluid in the CNS after neuronal injury (Figure 7A,B). These conditions were selected based on their ability to induce necrosis/apoptosis that activates calpain and caspase to trigger events that mimic neurodegenerative diseases [25]. Okadaic acid (OA) is a potent inhibitor of phosphatase 1/2A, resulting in hyperphosphorylation of specific proteins including tau [27,28,29,30,31]. OA was added to test cellular hyperphosphorylated tau vulnerability for fragmentation by activated proteolytic enzymes.

Using the total tau DAKO antibody (a.a. 243–441), control lane showed a band detected at 54K (non-phosphorylated tau) and minor band around 60K (p-tau) (Figure 7A,B). As for the RZ3 (pThr-231) and PHF-1 (pSer396/pSer404) antibodies, control lane showed a band at 54K (monomeric p-tau) and a minor band at 102K with PHF-1 (Figure 7A,B). αII-spectrin antibody showed considerable levels of intact 240K band and minor levels of spectrin breakdown products (SBDP-150K/145K/120K), suggesting a healthy neuronal culture. Treatment with STS, A23187, and MTX for 16h showed significant reduction of intact tau-54K (*p* < 0.0001) as detected by total tau (DAKO, DA31, DA9), and p-tau antibodies including RZ3 and PHF-1 compared to control (Figure 7A,B). Probing with αII-spectrin, we observed a considerable loss of intact spectrin-240K, and an increase of calpain-mediated SBDP-150K/145K, with STS, A23187, and MTX treatments.

OA treatment caused a considerable increase of monomeric p-tau-54K (DAKO, RZ3, and PHF-1) and formation of a high molecular weight 102K band. Interestingly, treatment with OA followed by STS protected the complete loss of 54K (RZ3 and PHF-1), but not the 102K band (PHF-1) suggesting that tau is less vulnerable to proteolysis with OA-induced hyperphosphorylation (Figure 7A,B). When samples were probed with αII-spectrin, we observed both the SBDP150K/145K and SBDP-120K, suggesting calpain and caspase activation. Similar to OA→STS treatment, we observed with OA→MTX and OA→A23187 a cluster of low molecular weight fragments (tau-BDP-24K) with 16h treatment when probed with DAKO (Figure 7A,B). Thus, STS, A23187, and MTX treatment caused a neurodegenerative paradigm that involves a dual contribution of calpain-1 and caspase-3 with the phosphorylated tau being partially protected from proteolysis induced by the neurotoxic challenges.

With technological advances in mass spectrometry, conditioned media received attention as a source for discovering new primary cleavage sites for peptides processing, ectodomain, and regulated and neurotoxic proteolytic peptides. Therefore, conditioned media from CTX culture treated with necrotic and apoptotic agents were collected for analysis by nLC-ESI-MS/MS to search for tau proteolytic fragments released in response to neurotoxic challenges. 

**Figure 8:**
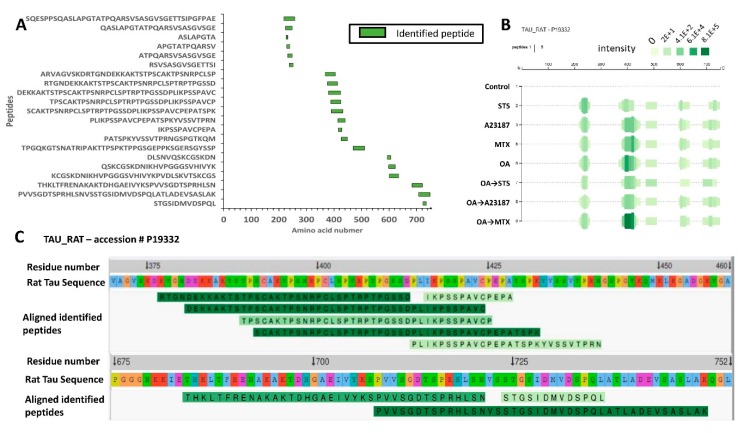
Identification of tau neuropeptides from conditioned media following neurotoxin challenges in rat cerebrocortical neurons. (**A**) Schematic representation for tau peptides generated and released into conditioned media. Peptides detected from control samples are not shown. Green box represents the identified peptide. (**B**) Peptide intensity profile of -rat Tau (accession #: P19332) digested by calpain-1. At each amino acid residue along the protein, the height of the green bars is proportional to the count of peptides overlapping this position. The intensity of the color (green) is proportional to the sum of the peptide intensities overlapping this position. (**C**) A customized Peptigram peptide alignment map compiled from rat tau-752. Peptides from the input data are highlighted in green. The intensity of each peak detected by mass spectrometry is represented by a green color gradient, the darker the green color the higher the intensity. This alignment is generated from tau-752 starting from the N-terminal domain: a.a. 375–752.

Cells within a biological system secrete tau as full-length or proteolytic fragments, depending on cellular environments [32]. Table 4 shows the list of peptides identified from CTX culture conditioned media. We did not observe rat N-terminal tau peptides in our analysis (Figure 8A). All peptide clusters were identified starting around an a.a. 200–700 range on the rat tau isoform (accession # P19332). One of interest is the C-terminal peptide, PRHLSNVSSTGSIDMVDSPQLA (a.a. 716–737; accession number P10637), corresponding to the amino acid 405–426 on human tau-441 isoform, which we have seen with our in vitro experiment with tau (Figure 6B,C). A cluster of similar peptides was observed in htau mouse brain digested by calpain-1. The presence of the LMW C-terminal tau fragments has been reported in a study showing that synaptosomes from AD brains had high levels of tau fragments (20–22K), with the majority lacking the C-terminal domain [6]. Our peak intensity analysis overlaps with the PSM counts showing high peptide intensity around a.a. residue 210–250, 400–450, and a.a. 700–737 of rat tau (Figure 8B). We did not observe peptide released with high confidence from control or OA treatment for 16h (Figure S3A). One might expect such an outcome as OA treatment causes hyperphosphorylation of tau, making it less vulnerable to proteolysis. 

With STS treatment, we observed the highest number of hits of proteolytic tau peptides when compared to other tested conditions (Figure S3A). One peptide observed in our in vitro digestion is STGSIDMVDSPQL (a.a.724–736), with a phosphorylation site probability of 99% at Ser724 residue (corresponding to pSer413 on human tau441) (Figure S3B).

STS is a pan-kinase inhibitor that is known to induce apoptosis by activating caspase-3 [33]. Others and we have identified a.a. 413–414 as caspase-3 cleavage site on the human tau-441, suggesting the involvement of caspase-3 in the generation of this fragment [6,25]. When cells were pretreated with OA followed by STS, we observed unique fragments not found with STS treatment alone (Figure S3A). As for A23187, no high confidence fragments were detected with and without OA pretreatment (*n* = 3). An explanation might be that A23187 did not produce LMW (<5K) fragments released into the conditioned media during the 16h treatment; therefore, longer time-course treatment time is needed to confirm this observation. MTX (with and without OA) treatment resulted in peptides of varying length ranging starting from a.a. 377–441 on the rat tau-752 (Figure S3A).

### 2.4. Limitation of the Current Study 

In this study, we analyzed samples from recombinant human tau, a transgenic mouse with htau, and rat primary cerebrocortical culture. The use of rat neuronal culture might provide valuable data for the identification of tau proteolytic peptides and studying other neurodegenerative processes. Rodents were chosen to prepare primary culture due to accessibility, reduced expenses, and less strict regulations. However, rodents do not usually encounter human-like neurodegenerative disorders like AD or Parkinson’s disease (PD).

Additionally, calpain might process rat tau isoform different from human tau isoform producing different cleavage sites. Differences between rat and animal tau sequences may influence calpain cleavage sites. A few studies have indicated using human primary fetal neuron cultures [34,35]. There continues, however, a shortage of journals outlining the development of human neuronal culture. Current techniques to differentiate pluripotent stem cells (iPSCs) into human-induced neurons (iNS) offer an exciting opportunity to explore tau proteolysis in human neurons [36].

## 3. Conclusions and Future Directions 

In this study, we used in vitro analysis of calpain-mediated tau fragments as a template to mimic the proteolysis of tau after neurodegeneration. We can summarize from our immunological and peptidomic studies that calpain-1 produced mainly distinct N-terminal (aa 2–19) and C-terminal peptides (413–441) with varying lengths from purified tau, mouse brain lysate, and culture-based neurotoxic challenges. The approach of peptidome has been used in other studies as a possible source of identifying novel biomarkers from neurodegenerative diseases. To our knowledge, this is the first systematic approach to characterize calpain-mediated proteolytic fragments of tau for discovering biomarkers using nano-LC-MS/MS. This could serve for clinical diagnosis and therapy approach defined as “theranostic”. Our overall data in this study shows that tau is not only degraded into large molecular weight fragments by calpain, but also into fragments which are low in molecular weight peptides that could simultaneously play a role in the neurodegenerative diseases such as Alzheimer’s disease, schizophrenia, CTE, and TBI. Our results were also consistent with previous studies that showed the high vulnerability of tau to calpain, producing break down products of varying length depending on the stage on the injury [25]. 

In a future study, we aim to use our peptidomic approach to verify the identified tau proteolytic peptides and brain protein-breakdown products in tauopathy neurodegenerative disease models (e.g., TBI and CTE) and human tauopathy bio-fluid samples (e.g., blood, serum, and cerebrospinal fluid (CSF)). We intend to approach the analysis of human bio-fluid using a translational strategy developing an optimized multiple reaction monitoring (MRM) quantification method in MS/MS of the tau brain proteolytic peptides. For validation in the bio-fluid samples, we will implement an antibody-based competitive enzyme-linked immunosorbent assay (ELISA) to target the peptide epitope region of interest. Additionally, the experiments from this study can be repeated on a transgenic mouse with htau using neurotoxic agents (e.g., NMDA, A23187) to characterize tau proteolytic peptides. 

Thus, these results may be used in parallel in clinical studies to monitor the activity of calpain to investigate if similar patterns of tau peptides are affected in different disease or to monitor the effect of pharmaceutical drugs.

## 4. Materials and Methods

### 4.1. Mouse Brain Collection and Samples Preparation

In our study, all mice used aged three months. For naïve transgenic human tau mouse brain samples, mice were anesthetized and killed by decapitation. Subsequently, the cortex and hippocampus part of the brains were removed and flash-frozen in liquid nitrogen. The brain samples were pulverized to a fine powder using a mortar and a pestle set in dry ice and chilled with liquid nitrogen. The fine powder was then transferred to microcentrifuge tubes (Eppendorf, Germany). The brain powder was lysed with 1% Triton X-100 lysis buffer containing 20 mM Tris HCl, pH 7.0, five millimolar ethylenediamine tetraacetic acid (EDTA; Sigma, St. Louis, MO, USA), and one millimolar dithiothreitol (DTT) in LC-MS grade water. The brain lysates were incubated at 4 °C for 120 min kept at a tube revolver (Thermo scientific, Waltham, MA, USA) at low speed. Following the incubation, the samples were centrifuged at 10,000× *g* for 15 min at 4 °C, and the supernatant was transferred to new tubes. The protein concentration was determined by performing protein assay using bicinchoninic acid microprotein assays (Pierce Inc., Rockford, IL, USA) against albumin standards. The brain lysates were then stored at −80 °C until further use.

Ethics Statement: All animal studies conformed to the guidelines outlined in the ‘Guide for the Care and Use of Laboratory Animals’ from the National Institutes of Health and were approved by the University of Florida.

### 4.2. In Vitro Calpain-1 Digestion of Purified Tau and Naïve Mouse Brain Lysate

Purified recombinant human tau-441 protein (non-phosphorylated and phosphorylated; five µg each; rPeptide^TM^) or mouse cortex naïve brain lysate (10 µg) were used for the study. The reaction buffer contained 1 mM of DTT (Sigma, St. Louis, MO, USA), 1 mM of CaCl_2_, and 100 mM Tris/HCl (pH 7.4). The samples were diluted with ultra-pure water for a final volume of 100 µL per reaction. The samples were incubated with calpain-1 (Enzo Life Sciences, Farmingdale, NY, USA; 1µg/µL) for 1 h at 30 °C followed by addition of 1µM of SNJ-1945 to stop the reaction (a calpain inhibitor; Senju Pharmaceutical Co. Ltd., Kobe, Japan). Calpain-1 was titrated in the ratio of 1:10, 1:25, 1:50, 1:100, and 1:250 (enzyme to protein ratio). The control contained only the purified protein or naïve mouse brain sample without the addition of calpain enzyme. 

### 4.3. Rat Primary Cerebrocortical Neuronal (CTX) Culture 

Rat primary cerebrocortical neuronal culture (CTX) were harvested from a homogenized pool of one-day-old Sprague–Dawley rat brains and were plated on poly-L-lysine-coated 12-well culture plates (Erie Scientific, Portsmouth, NH, USA) at a density of 4.36 × 10^5^ cells/mL. Cultures were maintained in Dulbecco’s modified Eagle’s medium (DMEM) with 10% fetal bovine serum in a humidified incubator in an atmosphere of 10% CO_2_ at 37 °C. After three days, the DMEM solution was replaced with another DMEM solution containing 1% cytosine arabinoside (ARC). Two days later, the solution was replaced with DMEM. The cells were cultured for an additional 10 days before use. Subsequent media changes were done twice a week.

### 4.4. Neurotoxic Challenges

Complete media was replaced with Serum-free medium, Neurobasal-A (Gibco, Waltham, MA, USA) consisting of 1% (v/v) B-27 supplement (Gibco), one millimolar L-glutamine (Sigma), and 1% (v/v) penicillin-streptomycin (Sigma) in a total volume of 500 microliters per well in a 12-well plate. In addition to untreated controls, the following conditions were used: apoptotic inducer STS (staurosporine; 0.5 µM; Sigma) that activate calpain and caspase-3, calcium ionophore A23187 (calcimycin; 20 µM; Sigma, St. Louis, MO, USA), and maitotoxin (MTX; 10 nM; Sigma) for 16 h. A23187 and MTX both acts by activating extracellular calcium channels leading to an increase of cytosolic Ca^2+^ ions, and indirectly can activate calpain-1 and -2. The conditions mentioned above were pretreated with or without okadaic acid (OA; 100 nM; Cell Signaling, Danvers, MA, USA) for six hours prior the additions of STS, A23187, and MTX challenges.

### 4.5. Preparation and Collection of Cell Lysate and Conditioned Media

After the treatment, conditioned media were collected from each well, added into separate tubes on ice, and centrifuged at 10,000× *g* for 10 min at 4 °C. The supernatants were collected frozen at −80 °C until further analysis. As for the attached CTX cells, 100 µl per well of lysis buffer that included:1 mM DTT, 1× phosphatase inhibitors (Sigma), 1% Mini-complete protease inhibitor cocktail tablet (Roche Biochemicals), and 1% Triton X-100 (Sigma) was added. The attached cells were then scraped down into the lysis buffer and collected into a separate 1.5 mL Eppendorf tubes. The cell lysates were incubated for 90 min at 4 °C and then centrifuged at 15,000 rpm for 15 min to remove cell debris. The supernatants were collected and frozen at −80 °C until analyzed by SDS-PAGE followed by western blotting.

### 4.6. Sample Preparation and Ultrafiltration 

The calpain-1 digested purified tau protein, mouse brain lysate, and CTX conditioned cell media were loaded into 10K molecular weight cut-off (MWCO) membrane filter (Vivacon 500 HY, Sartorius Stedim Biotech, Goettingen, Germany) and centrifuged at 3000× *g* at 4 °C for 60 min. Each condition was prepared in three biological replicates (*n* = 3). The filtrates were evaporated until dryness using speed vacuum (Thermo Scientific) and reconstituted in 20 µL of LC-MS grade 0.1% formic acid in water. The samples were stored at −80 °C pending analysis. The retentate fractions were saved for SDS-PAGE described in the following section. As for the cell-conditioned media from CTX culture, the same protocol mentioned above was followed except subjecting the filtrates to ZipTip cleanup (Millipore Sigma) before nLC-ESI-MS/MS analysis. The ZipTip C_18_ (0.6 μL bed volume; 5 μg) were conditioned with 3× 10 μL of 100% ACN and equilibrated by 3× 10 μL of 0.1% formic acid (FA), after which the samples were aspirated ten times for maximum binding. The samples were then washed 1× 10 μL 0.1% FA and subsequently eluted with ten microliters of 70% ACN, 0.1% FA. Finally, the eluate solvents were evaporated in speed vacuumed until dryness and stored at −80 °C. The samples were re-suspended in 10 μL 0.1% FA.

### 4.7. Gel Electrophoresis and Western Blotting

Equal amounts of purified protein (5 μg), CTX lysate and mouse brain lysate proteins (20 μg) were prepared for SDS–PAGE in 8 × Laemli loading buffer containing 0.25 M Tris (pH 6.8), 5% beta-mercaptoethanol (BME), 8% SDS and 0.02% bromophenol blue. Samples were subjected to SDS-PAGE on 4–20% precast-gels for 60 min at a constant voltage of 200V. After separation, the gels were transferred on to polyvinylidene difluoride (PVDF) membranes using iBlot transfer (Invitrogen, Life Technologies).

The membranes were blocked in 5% milk prepared in tris buffered saline with tween (TBST) for 1 h. After blocking, the membranes were incubated at 4 °C overnight with continuous shaking with primary antibodies. Primary antibodies used include PHF-1, CP-13, RZ3 (a gift from Peter Davies, Albert Einstein College of Medicine, Bronx, NY, USA), and total tau (DA9 and DAKO). αII-spectrin antibody (BML-FG6090, ENZO Life Sciences, Farmingdale, NY, USA) was used to assess cell injury.

β-actin and UCH-L1 antibody were used as a loading control. After overnight primary antibody incubation, membranes were washed three times with TBST and probed using anti-rabbit or anti-mouse immunoglobulin G (IgG) conjugated to alkaline phosphatase (Amersham, Piscataway, NJ, USA), for 1 h at room temperature and Immunoreactive bands were detected by developing with nitro blue tetrazolium, and 5-bromo-4-chloro- 3-indolyl phosphate (BCIT/NBT) (KPL, Gaithersburg, MD, USA). A 250K to 14K-rainbow molecular weight marker (RPN800E, GE Healthcare, Biosciences, Pittsburgh, PA, USA) was used to estimate the molecular weight of the proteins. Quantitative evaluation of protein levels was performed via image-J software (developed by National Institute of Health (NIH))). 

### 4.8. Nano-LC-ESI-MS/MS

The filtrate fractions were vortexed for 15 s and centrifuged at 15,000 rpm for 2 min. This step was repeated twice to make sure all the residues has been dissolved. The samples were transferred into auto-sampler micro vial tubes avoiding any air-bubbles. Five microliters of each sample were injected into the nano-LC system (NanoAcquity, Waters Corp, Milford, MA, USA)). The separation column used was C_18_ (130Å, 1.7 µm, 100 μm × 100 mm; Waters), fitted with a C_18_ trap column (5 µm Symmetry 180 µm × 20 mm), and coupled to linear ion trap mass spectrometry (LTQ-XL; Thermo Scientific, Waltham, MA, USA). The flow rate was set to 300 nL/min. Peptide separation was achieved using a 120 min linear-gradient, running from 1 to 50% of solvent B (0.1% formic acid in 100% acetonitrile). Tandem mass spectrometry was performed on LTQ-XL (Thermo, San Jose, CA, USA). The mass spectrometer was calibrated using the positive ion standard calibration solution (Thermo) to maintain the accuracy of the instrument. The peptide samples eluted from the reversed-phase column were introduced into the mass spectrometer using a nano-spray ionization source (ADPC-IMC adaptor, New Objective, Woburn, MA, USA). The mass spectrometer was operated in a data-dependent acquisition mode with a spray voltage of 2.1 kV, ion transfer tube voltage at 35 V, and an ion transfer tube temperature at 275 °C. The fragmentation mode was set to collision-induced dissociation (CID) with Helium gas as the collision gas.

The sheath and auxiliary gases were set to zero. The ion signal threshold was set to 1000 for MS/MS. The normalized collision energy was set to 35%, activation of q = 0.25, and activation time of 30 ms for MS/MS acquisitions. The instrument setting for MS scan was: m/z 300–1800, max injection time 200 ms; automatic gain control (AGC) target 1e6. Data-dependent acquisition mode with automatic switching between MS and MS/MS modes was employed. CID MS/MS was set to collect the top ten most abundant ions for fragmentation. The isolation width was set to m/z 1.5. The CID target value was set to 10,000 ions for fragmentation. The following dynamic exclusion setting was applied to precursor ions chosen for MS/MS analysis: repeat count—1; repeat duration—30 s; and exclusion duration—120 s. The neutral loss experiment where data-dependent settings were chosen to trigger an MS3 scan when a neutral loss of 97.97, 48.99, or 32.66 *m*/*z* units (corresponding to singly, doubly, or triply charged phosphorylated daughter mass ions, respectively) was detected among the most abundant 10 product-ions to enhance the fragmentation of phosphopeptides. Multistage activation feature was enabled on X caliber 2.4.1 for neutral loss detection. Each analysis was repeated three times to confirm the reliability of the mass spectrometric analysis.

### 4.9. Thermo Orbitrap Fusion Tribrid Mass Spectrometer Analysis

Filtrate peptides were desalted with C_18_ solid-phase extraction according to manufacturer protocol. For high-resolution analysis, we used a Thermo Orbitrap Fusion tribrid mass spectrometer with high energy collisional dissociation (HCD) in each MS and MS/MS cycle (Thermo Scientific, Bremen, Germany). The instrument was run in a data-dependent mode with a full MS (*m*/*z* 400–2000) resolution of 70,000 and 10 MS/MS experiments (HCD-normalized collision energy (NCE) = 28%, isolation width = 3 Th, first mass = 105 Th, 5% underfill ratio, peptide match set to “preferred”, and an AGC target of 1e6). Dynamic exclusion for 10 s was used to prevent repeated analysis of the same peptides, and a lock mass of *m*/*z* 445.12003 (polysiloxane ion) was used for real-time internal calibration. The MS system was interfaced with an automated Easy-nLC 1000 system (Thermo Fisher Scientific, Bremen, Germany). Each sample fraction was loaded onto an Acclaim Pepmap 100 precolumn (20 mm × 75 µm; 3 µm-C18) and separated on an Easy-Spray analytical column (500 mm × 75 µm; 2 µm-C18) at a flow rate at 300 nL/min during a linear gradient from solvent A (0.1% formic acid (v/v)) to 25% solvent B (0.1% formic acid (v/v), 99.9% acetonitrile (v/v)) for 280 min, followed by ramping up to 98% solvent B for an additional 20 min. Peptides were sprayed into the orifice of the mass spectrometer, which was operated in an information-dependent data acquisition mode.

### 4.10. Data Analysis

The MS/MS data analysis was done using Proteome Discoverer 2.2. (Thermo Scientific, Bremen, Germany). The MS and MS/MS spectra were searched against mouse tau (UniProt Identifier: P10637), rat tau (Identifier: P19332) and human (Identifier: P10636) tau isoforms (all accessed Sept 2017 from UniProtKB/Swiss-Prot) using their Fasta file through Sequest HT 2.5.0 search engine. Since we selected non-specific enzymatic digestion in Sequest HT, it would take a longer time to analyze the whole rat, mouse, and human proteome database. Therefore, we created a subset database that includes only tau isoforms from the mentioned species above. To validate that the identified peptides are only present in tau and not another protein, we searched the raw files using the protein BLAST database. 

The minimum and the maximum peptide length were set to 6 and 30, respectively. The search was performed with no enzyme specificity; oxidized methionine; phosphorylated serine, threonine, and tyrosine (+79.9663 Da) as dynamic modifications. For the LTQ-XL instrument, the precursor mass error tolerance was set to 1.5 Da, and product-ion tolerance was set to 0.8 Da. 

For the Orbitrap Fusion Tribrid instrument, the precursor mass error tolerance was set to 10 ppm, and daughter ion tolerance was set to 0.6 Da. The tandem mass spectra and product-ions were inspected manually to make sure the quality of the CID fragmentation and phosphorylation site identified. The following peptide scores selected were: ΔCn > 0.1, PSM > 20 and XCorr > 2. A target FDR value of 1% was set for decoy searches. The phosphorylation sites probabilities were assigned using the post-translational modification (ptmRS) node on Proteome Discoverer 2.2, which localize phosphorylation sites based on the search engine identification. 

### 4.11. Statistical Analysis

Statistical analysis was performed GraphPad Prism 7.0 (GraphPad, La Jolla, CA) with a one-way ANOVA Tukey’s Test. For multiple comparisons, a one-way ANOVA followed by Bonferroni’s post-hoc test was performed. * *p* < 0.05, ** *p* < 0.01, *** *p* < 0.001, **** *p* < 0.0001, ns: non-significant. Peptides presented had at least an XCorr value of 2, two PSM, and high confidence to be included for the analysis. Peptides with *p*-values < 0.05 were reported.

## Figures and Tables

**Table 1:** Selected human tau-441 calpain-mediated proteolytic peptides from purified protein digestion identified by nano-liquid chromatography-tandem mass spectrometry (nLC-MS/MS).

Annotated Sequence	#PSMs	Positions	Theo.MH+ [Da]	a.a.	Charge	m/z [Da]	XCorr
AEPRQEFEVMEDHAGTYG	65	2–19	2065.89	18	3	689.62	4.71
LSKVTSKCGSLG	46	315–326	1179.64	12	1	1179.61	3.73
SPRHLSNVSSTGSIDMVDSPQLA	81	404–426	2398.17	23	2	1199.91	5.69
TLADEVSASLAKQGL	37	427–441	1502.81	15	3	502.35	4.47
SASLAKQGL	32	434–441	787.467	8	2	394.37	2.51
SPRHLSNVSSTGSIDMVDSPQLATLADEVS	29	404–433	3113.51	30	3	1038.75	5.35
KAKTDHGAEIVYKSPVVSGDT	28	383–403	2202.14	21	3	735.03	4.05
RENAKAKTDHGAEIVYKSPVVSGDT	26	379–403	2672.36	25	3	891.93	5.3
AEPRQEFEVMEDHAGTYGLG	24	2–21	2236.31	20	2	1118.87	4.05

Note: a.a. stand for amino acids length; PSM: peptide sequence matches; Theo. MH+ stand for theoretical mass in Daltons. All these measurements are obtained from proteome discoverer 2.2.

**Table 2:** Selected human phosphorylated tau-441 calpain-mediated proteolytic peptides from purified protein digestion identified by nLC-MS/MS.

Annotated Sequence	PSMs	Positions in Proteins	Theo. MH+ [Da]	Sequence Length	*m*/*z* [Da]	XCorr
STGSIDMVDSPQLA	77	413–426	1500.63	14	751.098	4.18
KSPVVSGDTSPRHLSNVS	17	395–412	2106.87	18	1054.21	5.4
STGSIDMVDSPQL	16	413–425	1349.63	13	675.64	3.98
KPVDLSKVTSKCGSLG	7	311–326	1618.88	16	810.24	3.69
AAAQPHTEIPEGTTAEEAGIGDTPSLEDEAAGHVT	4	89–123	3472.56	35	1158.62	6.22
KPVDLSKVTSKCG	2	311–323	1361.75	13	681.41	3.44
KSPVVSGDTSPRHLSNVSSTGSIDMVDSPQLA	2	395–426	3508.51	32	1170.50	5.01

Note: a.a. stand for amino acids length; PSM: peptide sequence matches; Theo. MH+ stand for theoretical mass in Daltons. All these measurements are obtained from proteome discoverer 2.2.

**Table 3:** Selected tau-441 calpain-mediated proteolytic peptides from mouse brain lysate digestion identified by nLC-MS/MS.

Annotated Sequence	PSMs	Positions in Proteins	Theo. MH+ [Da]	Sequence Length	Charge	*m*/*z* [Da]	XCorr
TLADEVSASLAKQGL	9	427–441	1502.80	15	2	752.17	3.5
STGSIDMVDSPQLA	6	413–426	1420.66	14	1	1420.76	2.83
LSKVTSKCGSLG	4	315–326	1179.64	12	2	590.70	2.89
TDHGAEIVYKSPVVSGDT	4	386–403	1874.91	18	2	938.46	2.9
SPRHLSNVSSTGSIDMVDSPQLATLADEVS	3	404–433	3113.50	30	3	1039.01	4.38
ESPLQTPTEDGSEEPGSETSDAK	3	45–67	2391.03	23	2	1196.55	3.55
SPRHLSNVSSTGSIDMVDSPQLA	3	404–426	2398.16	23	2	1199.91	4.53
ASLAKQGL	2	434–441	787.46	8	2	394.57	2.15
TPPKSPSSAKSRLQT	2	231–24	1584.87	15	2	793.316	2.53
NIHHKPGGGQVEVKSEKLDFKDRVQ	2	327–351	2845.50	25	3	949.78	3.86

Note: a.a. stand for amino acids length; PSM: peptide sequence matches; Theo. MH+ stand for theoretical mass in Daltons. All these measurements are obtained from proteome discoverer 2.2.

**Table 4:** Selected rat tau-733 peptides from rat primary cultures conditioned media identified by nLC-MS/MS.

Sequence	PSMs	Positions	Sequence Length	Charge	*m*/*z* [Da]	XCorr
PRHLSNVSSTGSIDMVDSPQLA	55	716–737	22	3	849.14	3.5
RSVSASGVSGETTSI	39	238–252	15	1	1677.78	2.07
VSASGVSGETTSIPGF	25	240–255	16	1	1736.90	2.09
FSKVSAETQASPPEGPG	16	268–284	17	1	1767.22	2.07
HLSNVSSTGSIDMV	15	718–731	14	1	1685.57	2.26
DLSNVQSKCGSKDN	12	594–607	14	2	868.42	2.77
AKTTPSPKTPPGSGEPPKSGERSGYSSPGSPGTPGSRSRTPSLPT	8	484–528	45	3	1552.74	4.33
SSQESPPSQASLAPGTATPQARSVSASGVSGETT	7	217–250	34	2	1734.38	2.53
HKLTFRENAKAKTDHGAEIVYKSPVVSGDTSPRHLSNV	6	685–722	38	3	1476.76	3.85
TRIPAKTTPSPKTPPGSGEPPKSGERSGYSSPGSPGTP	2	480–517	38	2	1995.92	2.7

Note: a.a. stand for amino acids length; PSM: peptide sequence matches; Theo. MH+ stand for theoretical mass in Daltons. All these measurements are obtained from proteome discoverer.

## Supplementary Materials

Supplementary materials can be found at https://www.mdpi.com/1422-0067/20/20/5213/s1.
