# Supplementary material for: Novel Peptidomic Approach for Identification of Low and High Molecular Weight Tauopathy Peptides Following Calpain Digestion, and Primary Culture Neurotoxic Challenges
Source: Int J Mol Sci. 2019 Oct 21;20(20):5213. doi: 10.3390/ijms20205213 (PMC6829287; doi:10.3390/ijms20205213)
Supplement: Supplementary file 1 [file ijms-20-05213-s001.pdf]

Supplementary Content

Novel Peptidomic Approach for Identification Of Low and High Molecular Weight Tauopathy Peptides Following Calpain Digestion, And Primary Culture Neurotoxic Challenges

Hamad Yadikar[1,2,4], *Connor Johnson*[1], *Niko Pafundi*[1], *Edwin Mousawasse*[1], *Lynn Nguyen*[1], *Isabel Torres*[1], *Milin Kurup*[1], *Zhihui Yang*[1], *Firas Kobeissy*[1,3], *Richard Yost*[4], *Kevin K. Wang** [1,5]

[1] Program for Neurotrauma, Neuroproteomics & Biomarkers Research, Departments of Emergency Medicine, Psychiatry, Neuroscience and Chemistry, University of Florida, Gainesville, FL32611, USA

[2]Department of Biological Sciences, Faculty of Science, Kuwait University, P.O. Box 5969, Safat 13060, Kuwait.

[3]Faculty of Medicine, American University of Beirut Medical Center, Beirut, Lebanon.

[4]Department of Chemistry, Chemistry Laboratory Building, University of Florida, Gainesville, FL 32611, USA.

[5]Brain Rehabilitation Research Center, Malcom Randall VA Medical Center, 1601 SW Archer Rd. Gainesville, FL 32608, USA.

*Correspondence:

Kevin Wang, kwang@ufl.edu ; phone: +1 (352) 328-7663

Hamad Yadikar, hamad.yadikar@ku.edu.kw; Phone: 352-900-9687

KEYWORDS: Tau proteolysis; Tau hyperphosphorylation; calpain; Label-free quantification; Peptidomics; biomarker discovery; Traumatic brain injury; Chronic Traumatic encephalopathy; ultrafiltration; cell-based calpain activation

Supplementary material

Supplementary Table 1 Human tau-441 calpain-mediated proteolytic peptides from purified protein digestion identified by nLC-MS/MS. (.xlsx)

Supplementary Table 2 Selected human phosphorylated tau-441 calpain-mediated proteolytic peptides from purified protein digestion identified by nLC-MS/MS. (.xlsx)

Supplementary Table 3 Tau-441 calpain-mediated proteolytic peptides from mouse brain lysate digestion identified by nLC-MS/MS. (.xlsx)

Supplementary Table 4 Selected rat tau-776 peptides identified from conditioned primary culture media by nLC-MS/MS. (.xlsx)

Supplementary Figure 1 Transgenic mouse brain lysate tau proteolysis treated with calpain-1

The immunoblot is showing tau vulnerability to calpain-1 digestion, forming p-Tau-BDP of 55, 42K and 12K. Total tau antibodies used are (**A**) DAKO. Phospho-Tau antibody used are (**B**) RZ3 (pThr231) and (**C**) CP13 (pSer202). (**D**) shows Coomassie stain of the mouse brain lysate SDS-PAGE gel.

Supplementary Figure 2 Immunoblotting of calpain-mediated digestion of recombinant tau, phosphorylated tau, and mouse brain lysate.

Immunoblots of recombinant tau, p-tau and mouse brain lysate calpain-digested samples (10 μg each) using total DA9 antibody (a.a. 102-145). Different tau immunoreactive bands are pointed out with arrows. Monomeric intact tau and p-tau (~63K), and tau-BDP (43K, 24K, and 12K). Calpain-1 was added to each sample except the control (1:50, 1:25, 1:10) for one hour, and the reaction was stopped with SNJ-1945 (calpain inhibitor).

Supplementary Figure 3 Composite summary of tau proteolytic peptides and phosphorylation sites from rat CTX conditioned cell media

(**A**) Spreadsheet showing representative proteolytic peptides identified rat CTX conditioned media. This table listing shows the confidence of each peptide in each fraction. Peptides not found are represented as white color. Green color represents high confidence. Yellow color represents medium confidence. Red represents low confidence. (**B**) Schematic representation showing the domains of rat tau protein (Accession# P19332) and location of phosphorylation sites identified and predicted calpain cleavage sites

derived from in this study as detected by nano-LC/MS/MS. The yellow circles at the top of the diagram are phosphorylation sites detected without OA, while the ones at the bottom are with the addition of OA.

Supplementary Figure 4 MS/MS spectrum of the tau peptide AEPRQEFEVMEDHAGTYG released from calpain-1 digestion

(A) MS/MS spectrum for the tau-441 peptide AEPRQEFEVMEDHAGTYG (amino acid residues 2-19), charge +3, monoisotopic *m/z* 689.625 Da, displaying the fragment ions for this peptide. **(B)** Identified b^+ and y^+ type ions for the tau peptide shown in red and blue identified from the database search results

Supplementary Figure 5 MS/MS spectrum of the tau peptide SPRHLSNVSSTGSIDMVDSPQLA released from calpain-1 digestion

(A) MS/MS spectrum for the tau-441 peptide SPRHLSNVSSTGSIDMVDSPQLA (amino acid residues 404-426), charge +2, monoisotopic *m/z* 1199.9Da, displaying the fragment ions for this peptide. **(B)** Identified b^+ and y^+ type ions for the tau peptide shown in red and blue identified from the database search results

Supplementary Figure 6 MS/MS spectrum of the phosphorylated tau peptide STGSIDMVDSPQLA released from calpain-1 digestion

(A) MS/MS spectrum for the phosphorylated tau-441 peptide STGSIDMVDSPQLA (amino acid residues 413-426), charge +3, monoisotopic *m/z* 751.0989 Da, displaying the fragment ions for this peptide. The phosphorylation losses are shown in yellow boxes of the b and y ions **(B)** Identified b^+ and y^+ type ions for the tau peptide shown in red and blue identified from the database search results

Supplementary Figure 7 MS/MS spectrum of the tau peptide DRKDQGGYTMHQDQEGDTDAGLK released from mouse brain lysate calpain-1 digestion

(A) MS/MS spectrum for the tau-441 peptide DRKDQGGYTMHQDQEGDTDAGLK (amino acid residues 22-44), charge +3, monoisotopic *m/z* 2566.47873 Da, displaying the fragment ions for this peptide. **(B)** Identified b^+ and y^+ type ions for the tau peptide shown in red and blue identified from the database search results

Supplementary Figure 8 MS/MS spectrum of the tau peptide TLADEVSASLAKQGL released from calpain-1 digestion

(A) MS/MS spectrum for the tau-441 peptide TLADEVSASLAKQGL (amino acid residues 427-441), charge +2, monoisotopic *m/z* 752.26 Da, displaying the fragment ions for this peptide. **(B)** Identified b^+ and y^+ type ions for the tau peptide shown in red and blue identified from the database search results

Supplementary Figure 9 MS/MS spectrum of the rat tau peptide PRHLSNVSSTGSIDMVDSPQLA released from conditioned cell media of primary cerebrocortical cultures

(A) MS/MS spectrum for the phosphorylated tau-776 peptide **PRHLSNVSSTGSIDMVDSPQLA** (amino acid residues 716-737), charge +3, monoisotopic *m/z* 849.14Da, displaying the fragment ions for this peptide. The phosphorylation losses are shown in yellow boxes of the b and y ions **(B)** Identified b^+ and y^+ type ions for the tau peptide shown in red and blue identified from the database search results.

1196 – hTau mouse (3 months)

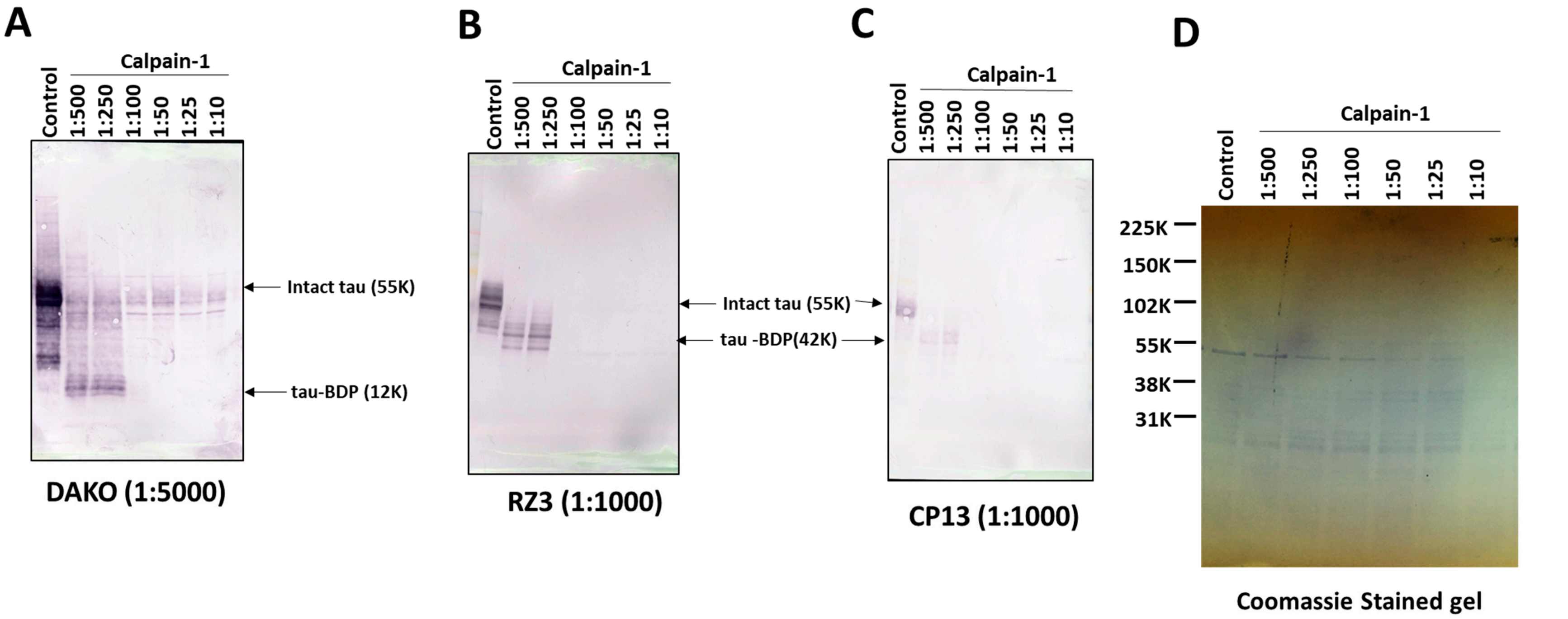

Supplementary Figure 1

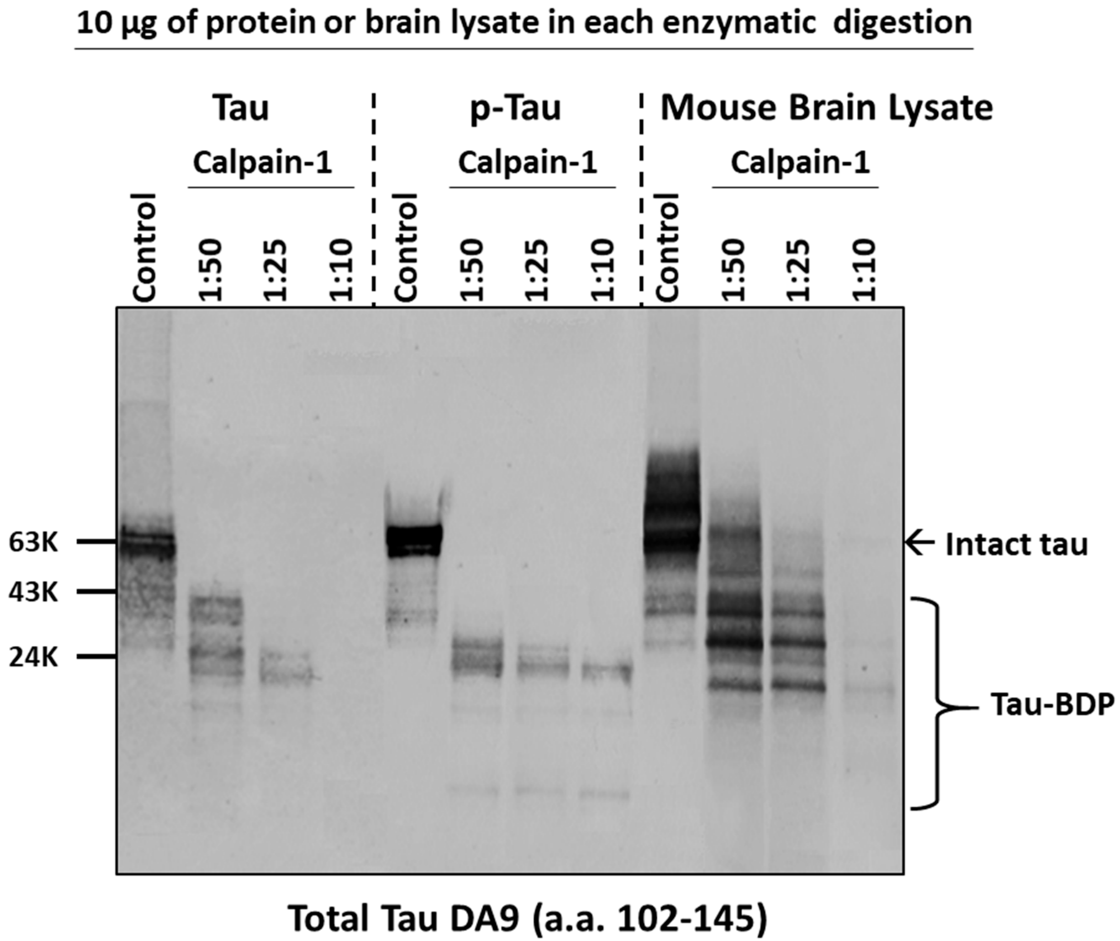

Supplementary Figure 2

A

Sequence	# PSMs	Positions in Prote	Control	STS	A23187	MTX	OA	OA→STS	OA→A23187	OA→MTX
SQESPPSQASLAPGTATPQARSVSASGVSGETTSIPGFPAE	18	P19332 [218-258]		High	Low	Low			Low	Low
ASLAPGTA	31	P19332 [226-233]		High	Low	Low		Low	Low	Low
ATPQARSVSASGVSGE	41	P19332 [233-248]		Low	Low	Low		High	Low	Low
RSVSASGVSGETTSI	58	P19332 [238-252]		Low	Low	Low		Low	Low	Low
ARVAGVSKDRTGNDEKKAKTSTPSCAKTPSNRPCLSP	3	P19332 [368-404]		High						
RTGNDEKKAKTSTPSCAKTPSNRPCLSPTRPTPGSSD	53	P19332 [377-413]			Low	Low		Low	Low	High
DEKKAKTSTPSCAKTPSNRPCLSPTRPTPGSSDPLIKPSSPAVC	54	P19332 [381-424]		Low	Low	Low			Low	High
TPSCAKTPSNRPCLSPTRPTPGSSDPLIKPSSPAVCP	80	P19332 [389-425]		Low	Low	Low		High	Low	Low
SCAKTPSNRPCLSPTRPTPGSSDPLIKPSSPAVCPEPATSPK	55	P19332 [391-432]			Low	Low		Low	Low	High
PLIKPSSPAVCPEPATSPKYVSSVTPRN	14	P19332 [414-441]			Low	High			Low	Low
IKPSSPAVCPEPA	14	P19332 [416-428]			Low	High		Low	Low	Low
PATSPKYVSSVTPRNGSPGTKQM	31	P19332 [427-449]			Low	Low		High	Low	
TPGQKGTSNATRIPAKTTPSPKTPPGSGEPPKSGERSGYSSP	46	P19332 [470-511]		Low		Low		High	Low	Low
KCGSKDNIKHVPGGGSVHIVYKPVDLSKVTSKCGS	30	P19332 [601-635]		High		Low		Low	Low	Low
THKLTFRENAKAKTDHGAEIVYKSPVVSGDTSPRHLSN	43	P19332 [684-721]				Low		High	Low	Low
STGSIDMVDSPQL	32	P19332 [724-736]		High		Low		Low	Low	Low

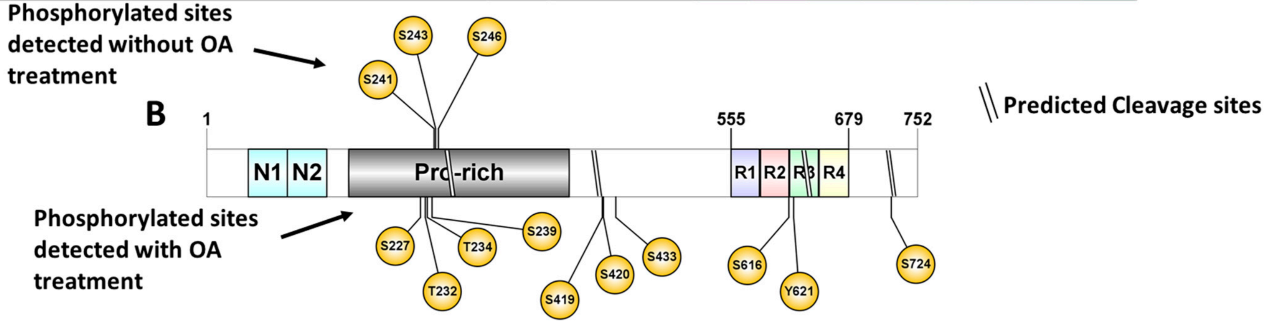

Supplementary Figure 3

Supplementary Figure 4

A

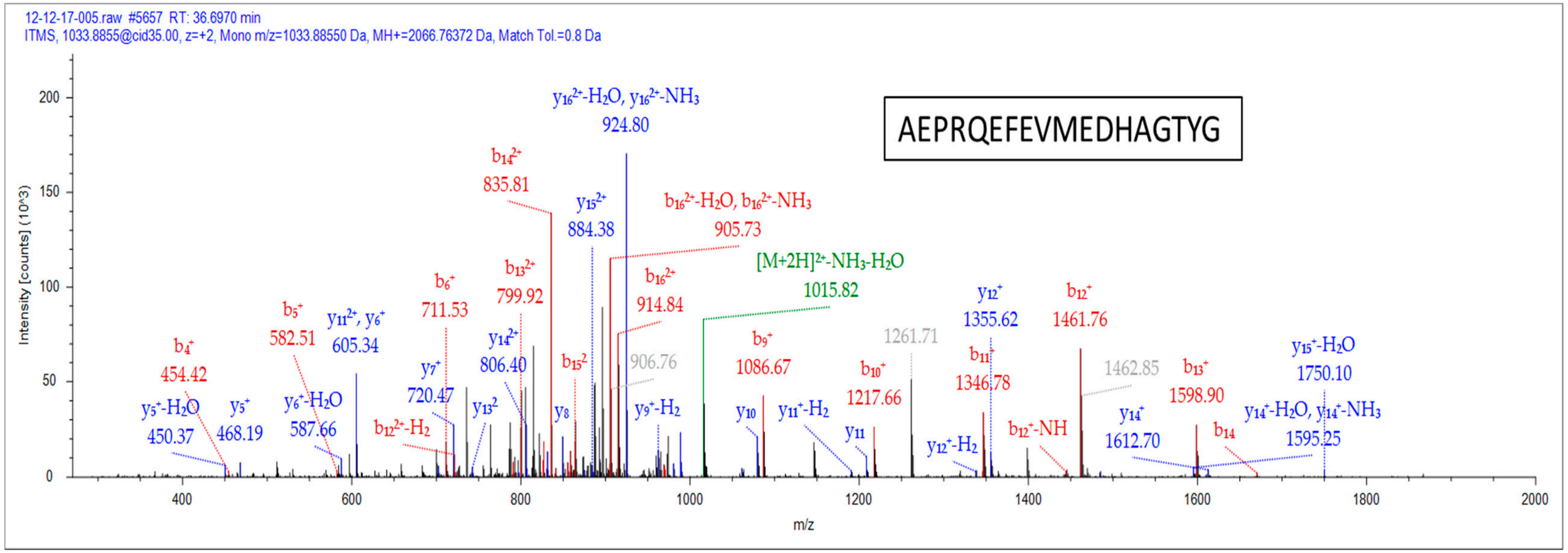

B

#1	b^{+}	b^{2+}	Seq.	y^{+}	y^{2+}	#2
b1	72.04	36.53	A			y18
b2	201.09	101.05	E	1994.85	997.93	y17
b3	298.14	149.57	P	1865.81	933.41	y16
b4	454.24	227.62	R	1768.76	884.88	y15
b5	582.30	291.65	Q	1612.66	806.83	y14
b6	711.34	356.17	E	1484.60	742.80	y13
b7	858.41	429.71	F	1355.56	678.28	y12
b8	987.45	494.23	E	1208.49	604.75	y11
b9	1086.52	543.76	V	1079.45	540.23	y10
b10	1217.56	609.28	M	980.38	490.69	y9
b11	1346.60	673.81	E	849.34	425.17	y8
b12	1461.63	731.32	D	720.29	360.65	y7
b13	1598.69	799.85	H	605.27	303.14	y6
b14	1669.73	835.37	A	468.21	234.61	y5
b15	1726.75	863.88	G	397.17	199.09	y4
b16	1827.80	914.40	T	340.15	170.58	y3
b17	1990.86	995.93	Y	239.10	120.05	y2
b18			G	76.04	38.52	y1

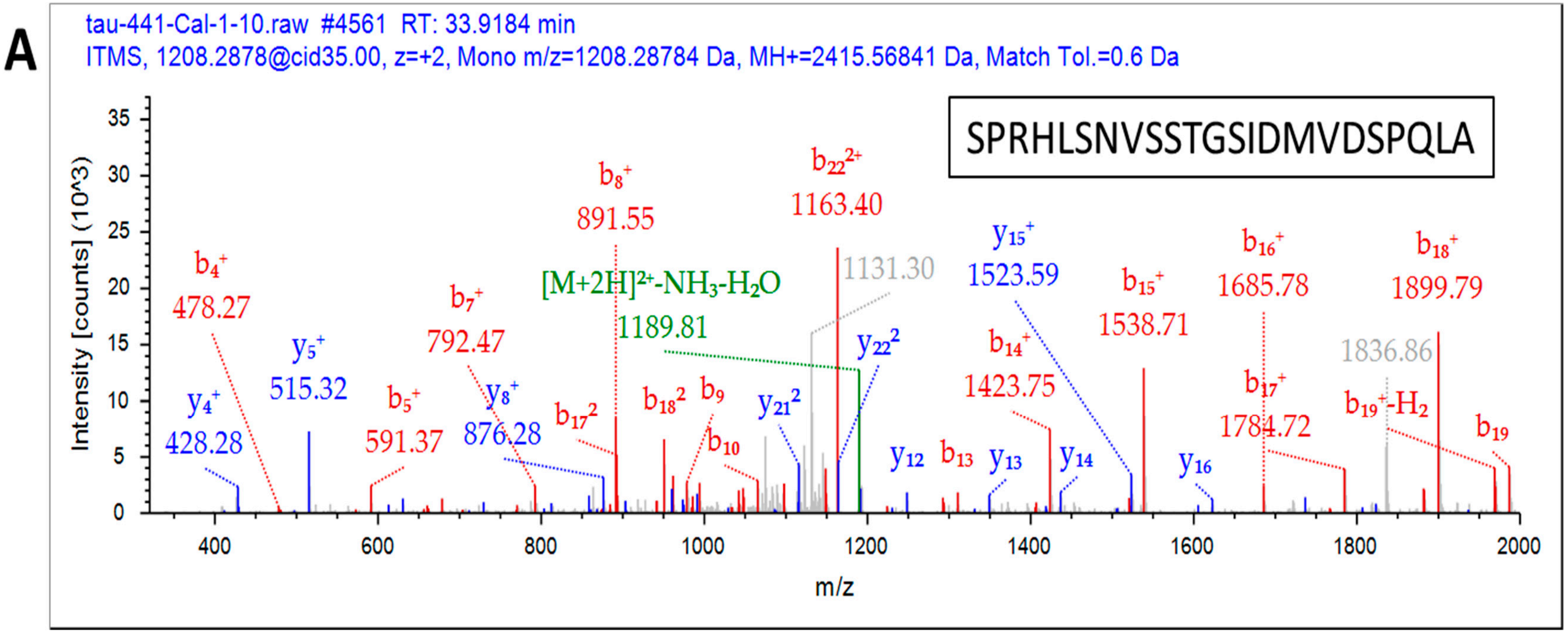

Supplementary Figure 5

Supplementary Figure 5

B

#1	b⁺	b²⁺	Seq.	y⁺	y²⁺	#2
b1	88.04	44.52	S			y23
b2	185.09	93.05	P	2327.13	1164.07	y22
b3	341.19	171.10	R	2230.08	1115.54	y21
b4	478.25	239.63	H	2073.98	1037.49	y20
b5	591.34	296.17	L	1936.92	968.96	y19
b6	678.37	339.69	S	1823.83	912.42	y18
b7	792.41	396.71	N	1736.80	868.90	y17
b8	891.48	446.24	V	1622.76	811.88	y16
b9	978.51	489.76	S	1523.69	762.35	y15
b10	1065.54	533.28	S	1436.66	718.83	y14
b11	1166.59	583.80	T	1349.63	675.32	y13
b12	1223.61	612.31	G	1248.58	624.79	y12
b13	1310.64	655.83	S	1191.56	596.28	y11
b14	1423.73	712.37	I	1104.52	552.77	y10
b15	1538.76	769.88	D	991.44	496.22	y9
b16	1685.79	843.40	M-Oxidation	876.41	438.71	y8
b17	1784.86	892.93	V	729.38	365.19	y7
b18	1899.89	950.45	D	630.31	315.66	y6
b19	1986.92	993.96	S	515.28	258.14	y5
b20	2083.97	1042.49	P	428.25	214.63	y4
b21	2212.03	1106.52	Q	331.20	166.10	y3
b22	2325.11	1163.06	L	203.14	102.07	y2
b23			A	90.05	45.53	y1

A

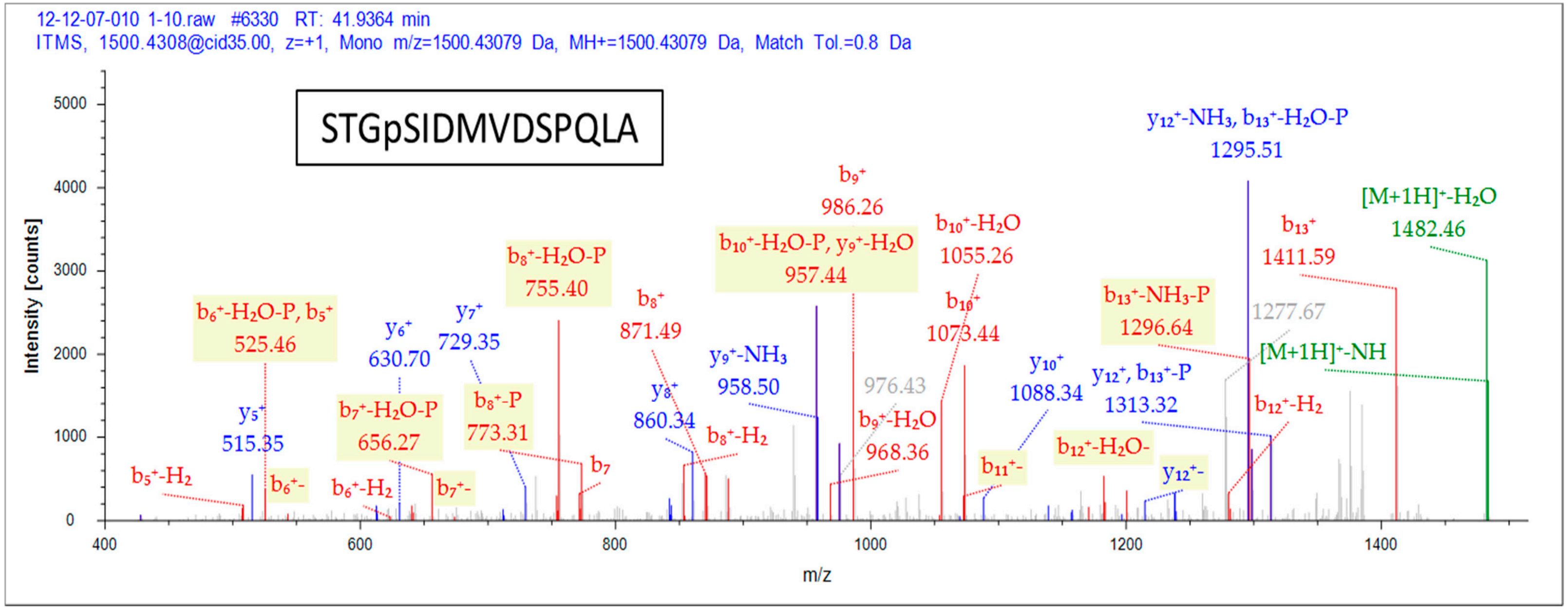

Supplementary Figure 6

B

#1	b^+	Seq.	y^+	#2
b1	88.04	S		y14
b2	189.09	T	1413.60	y13
b3	246.11	G	1312.55	y12
b4	413.11	S-Phospho	1255.53	y11
b5	526.19	I	1088.53	y10
b6	641.22	D	975.45	y9
b7	772.26	M	860.42	y8
b8	871.33	V	729.38	y7
b9	986.35	D	630.31	y6
b10	1073.39	S	515.28	y5
b11	1170.44	P	428.25	y4
b12	1298.50	Q	331.20	y3
b13	1411.58	L	203.14	y2
b14		A	90.05	y1

Supplementary Figure 6

A

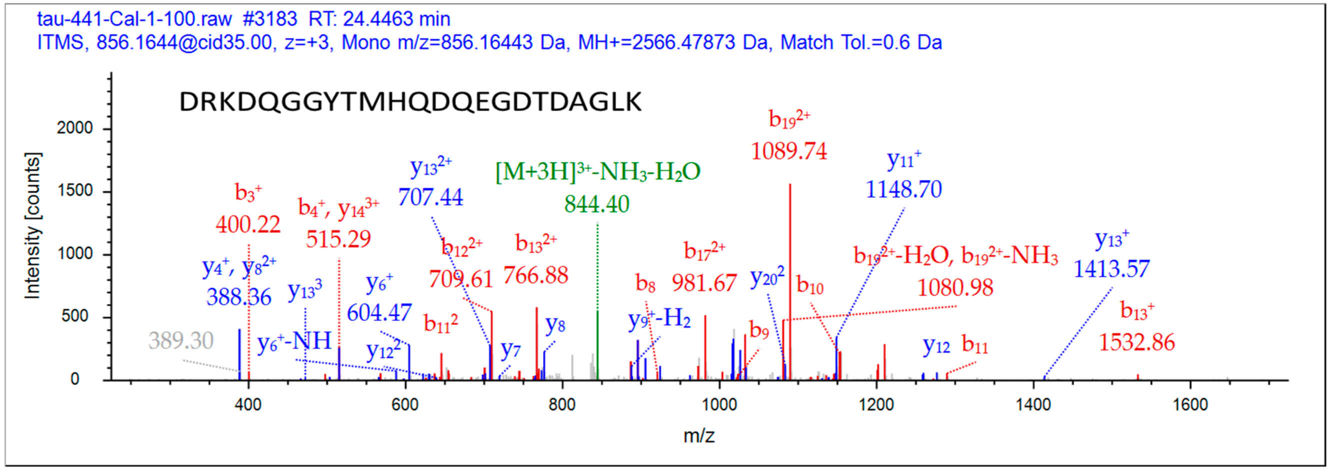

B

#1	b^{+}	b^{2+}	b^{3+}	Seq.	y^{+}	y^{2+}	y^{3+}	#2
1	116.03422	58.52075	39.34959	D				23
2	272.13533	136.57130	91.38329	R	2450.10009	1225.55368	817.37155	22
3	400.23029	200.61879	134.08162	K	2293.99898	1147.50313	765.33784	21
4	515.25724	258.13226	172.42393	D	2165.90402	1083.45565	722.63952	20
5	643.31581	322.16155	215.11012	Q	2050.87707	1025.94217	684.29721	19
6	700.33728	350.67228	234.11728	G	1922.81850	961.91289	641.61102	18
7	757.35874	379.18301	253.12443	G	1865.79703	933.40215	622.60386	17
8	920.42207	460.71467	307.47887	Y	1808.77557	904.89142	603.59671	16
9	1021.46975	511.23851	341.16143	T	1645.71224	823.35976	549.24226	15
10	1152.51023	576.75875	384.84160	M	1544.66456	772.83592	515.55970	14
11	1289.56914	645.28821	430.52790	H	1413.62408	707.31568	471.87954	13
12	1417.62772	709.31750	473.21409	Q	1276.56516	638.78622	426.19324	12
13	1532.65466	766.83097	511.55641	D	1148.50659	574.75693	383.50705	11
14	1660.71324	830.86026	554.24260	Q	1033.47964	517.24346	345.16473	10
15	1789.75584	895.38156	597.25680	E	905.42107	453.21417	302.47854	9
16	1846.77730	923.89229	616.26395	G	776.37847	388.69288	259.46434	8
17	1961.80424	981.40576	654.60627	D	719.35701	360.18214	240.45719	7
18	2062.85192	1031.92960	688.28882	T	604.33007	302.66867	202.11487	6
19	2177.87886	1089.44307	726.63114	D	503.28239	252.14483	168.43231	5
20	2248.91598	1124.96163	750.31018	A	388.25545	194.63136	130.09000	4
21	2305.93744	1153.47236	769.31733	G	317.21833	159.11280	106.41096	3
22	2419.02150	1210.01439	807.01202	L	260.19687	130.60207	87.40381	2
23				K	147.11280	74.06004	49.70912	1

Supplementary Figure 7

A

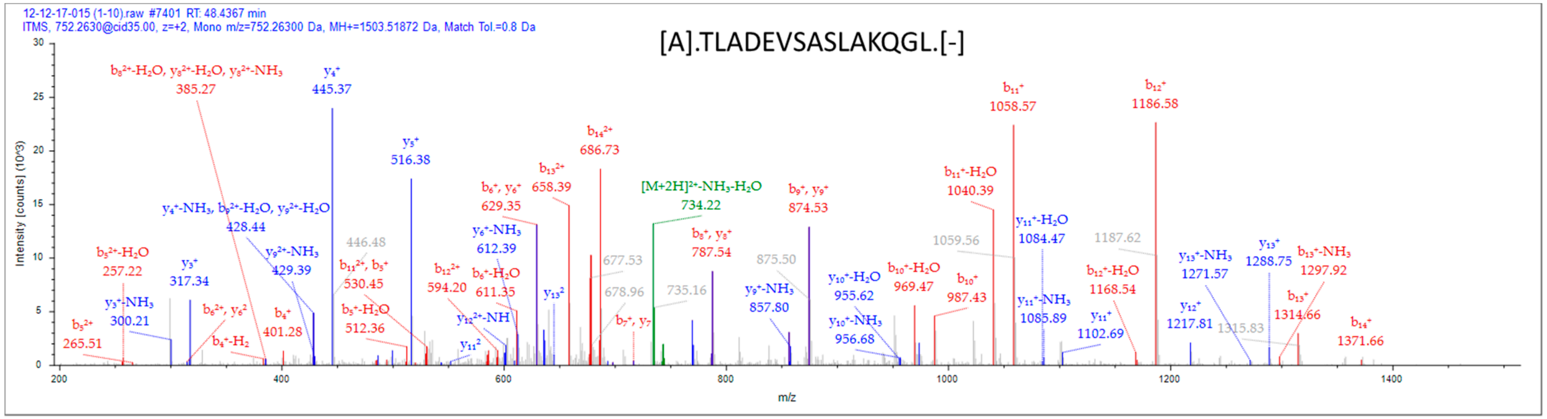

B

#1	b+	b2+	Seq.	y+	y2+	#2
1	102.05496	51.53112	T			15
2	215.13902	108.07315	L	1401.75838	701.38283	14
3	286.17613	143.59170	A	1288.67432	644.84080	13
4	401.20308	201.10518	D	1217.63721	609.32224	12
5	530.24567	265.62647	E	1102.61026	551.80877	11
6	629.31408	315.16068	V	973.56767	487.28747	10
7	716.34611	358.67669	S	874.49926	437.75327	9
8	787.38322	394.19525	A	787.46723	394.23725	8
9	874.41525	437.71126	S	716.43012	358.71870	7
10	987.49932	494.25330	L	629.39809	315.20268	6
11	1058.53643	529.77185	A	516.31402	258.66065	5
12	1186.63139	593.81934	K	445.27691	223.14209	4
13	1314.68997	657.84862	Q	317.18195	159.09461	3
14	1371.71143	686.35936	G	189.12337	95.06532	2
15			L	132.10191	66.55459	1

Supplementary Figure 8

A

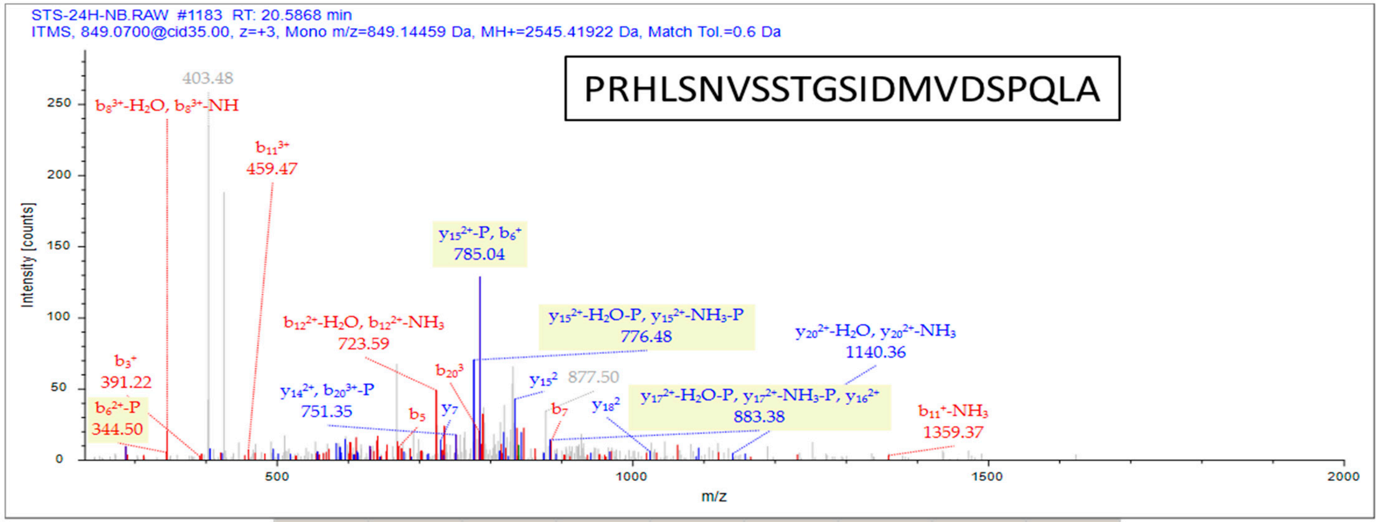

B

#1	b⁺	b²⁺	b³⁺	Seq.	y⁺	y²⁺	y³⁺	#2
b1	98.06	49.53	33.36	P				y22
b2	254.16	127.58	85.39	R	2453.98	1227.49	818.67	y21
b3	391.22	196.11	131.08	H	2297.88	1149.44	766.63	y20
b4	504.30	252.66	168.77	L	2160.82	1080.91	720.95	y19
b5	671.30	336.15	224.44	S-Phospho	2047.74	1024.37	683.25	y18
b6	785.35	393.18	262.45	N	1880.74	940.87	627.58	y17
b7	884.41	442.71	295.48	V	1766.70	883.85	589.57	y16
b8	1051.41	526.21	351.14	S-Phospho	1667.63	834.32	556.55	y15
b9	1218.41	609.71	406.81	S-Phospho	1500.63	750.82	500.88	y14
b10	1319.46	660.23	440.49	T	1333.63	667.32	445.21	y13
b11	1376.48	688.74	459.50	G	1232.58	616.80	411.53	y12
b12	1463.51	732.26	488.51	S	1175.56	588.28	392.53	y11
b13	1576.60	788.80	526.20	I	1088.53	544.77	363.51	y10
b14	1691.62	846.32	564.55	D	975.45	488.23	325.82	y9
b15	1822.66	911.84	608.23	M	860.42	430.71	287.48	y8
b16	1921.73	961.37	641.25	V	729.38	365.19	243.80	y7
b17	2036.76	1018.88	679.59	D	630.31	315.66	210.77	y6
b18	2123.79	1062.40	708.60	S	515.28	258.14	172.43	y5
b19	2220.84	1110.93	740.95	P	428.25	214.63	143.42	y4
b20	2348.90	1174.95	783.64	Q	331.20	166.10	111.07	y3
b21	2461.99	1231.50	821.33	L	203.14	102.07	68.38	y2
b22				A	90.05	45.53	30.69	y1

Supplementary Figure 9
